# Identifying recruitment strategies to improve the reach of evidence-based health promotion, disease prevention, and disease self-management interventions: a scoping review

**DOI:** 10.3389/fpubh.2025.1515042

**Published:** 2025-04-01

**Authors:** Paul A. Estabrooks, Mickey L. Bolyard, Tallie Casucci, Josh T. Christensen, Bryan Gibson, Caitlin A. Golden, Jennie L. Hill, Linnea Horvath, Shinduk Lee, Ellen M. Maxfield, Mary M. McFarland, James L. Merle, Tzeyu L. Michaud, Megan Miller, Emiliane L. Pereira, Chelsey R. Schlechter, Sara E. Simonsen, David W. Wetter, Amy B. Locke

**Affiliations:** ^1^Department of Family and Community Medicine, Spencer Fox Eccles School of Medicine, University of Utah, Salt Lake City, UT, United States; ^2^J. Willard Marriott Library, University of Utah, Salt Lake City, UT, United States; ^3^Faculty of Science, Brigham Young University – Hawaii, Laie, HI, United States; ^4^Department of Biomedical Informatics, School of Medicine, University of Utah, Salt Lake City, UT, United States; ^5^Department of Population Health Sciences, School of Medicine, University of Utah, Salt Lake City, UT, United States; ^6^College of Nursing, University of Utah, Salt Lake City, UT, United States; ^7^Osher Center for Integrative Health, University of Utah Health, Salt Lake City, UT, United States; ^8^Spencer S. Eccles Health Sciences Library, University of Utah, Salt Lake City, UT, United States; ^9^Department of Health Promotion, College of Public Health, University of Nebraska Medical Center, Omaha, NE, United States; ^10^Department of Population Health Sciences and Huntsman Cancer Institute, University of Utah, Salt Lake City, UT, United States

**Keywords:** dissemination science, individual-level dissemination strategies, participant engagement, participant identification, participation rate, representativeness

## Abstract

**Introduction:**

Improving the reach of existing lifestyle interventions focused on health promotion, disease prevention, and self-management delivered in community or clinical settings has the potential to increase the public health impact of these interventions. However, little is known about the overall success of recruitment strategies or the specification of strategy components including the details of how, through which channel, and by whom the recruitment strategies are enacted.

**Methods:**

We conducted a scoping review with guidance from the JBI Manual for Evidence Synthesis. For transparency and reproducibility, we adhered to the PRISMA-S and PRISMA-ScR guidelines for reporting literature searches and scoping reviews. Our eligibility criteria included studies that reported recruitment strategies to improve reach (enrollment number, participation rate, and representativeness of participants) of health promotion, disease prevention, and self-management lifestyle interventions for children or adults worldwide. Recruitment strategies for non-lifestyle interventions, such as pharmaceutical trials, were excluded. Databases included Medline (Ovid), Embase (embase.com), CINAHL Complete (Ebscohost), APA PsycINFO (Ebscohost), and Dissertation and Theses Global (ProQuest). Database search results were retrieved on March 2–3, 2023.

**Results:**

From a total of 9,712 references, 98 studies were included. Eight studies compared recruitment strategies using a randomized controlled trial and 90 studies were evaluations/quasi-experiments that reported on reach. There was a wide variety of recruitment strategies used, with 32% of the studies utilizing more than one recruitment strategy. The average reach, operationalized as participation rate, of the primary strategy (n = 15 defined strategies) being tested ranged from 3 to 41%. Further, participation rates ranged across studies that focused on children (43%), adults (25%), and older adults (16%). Most included studies did not report (1) strategy timing and dose, (2) theoretical basis, or (3) potential mechanisms of improved reach. Finally, differences in how the denominator was operationalized reduced confidence in comparing across strategies.

**Discussion:**

More clarity is needed when reporting on specific recruitment strategies used to improve the reach of lifestyle interventions. Suggestions include guidance on how to consistently define a denominator of eligible participants exposed to recruitment strategies. Furthermore, the use of theoretical approaches and testing of potential mechanisms of effect are needed in future studies to advance the science of improving lifestyle intervention reach.

**Systematic review registration:**

The unique identifier for our scoping review is 3g68b it can be found at this url: https://doi.org/10.17605/OSF.IO/3G68B.

## Introduction

There is a long history of developing and testing health promotion, disease prevention, and disease self-management (secondary prevention) interventions with the intent to have a public health impact (1, 2). As a result there are a myriad of efficacious interventions across these areas that have been compiled into registries to support broad dissemination and implementation (3). However, to achieve a public health impact, there is a need for these interventions to have broad reach and be effective. Reach is an individual-level dissemination outcome and can be defined as the number of participants that enroll, the proportion of eligible people exposed to recruitment activities that enroll, and the representativeness of those enrolled in a given health promotion intervention relative to the intended audience based on demographic characteristics (4). Further underscoring the need to address representativeness, public health goals also focus on increasing the reach of evidence-based interventions in populations that experience health disparities (5).

Within the field of dissemination and implementation science—where understanding the reach, adoption, implementation, and sustainment of evidence-based interventions is foundational—strategies that focus on improving the reach of evidence-based programs for all populations have increased in importance (5, 6). Unfortunately, over the previous 20 years, the degree to which intervention trials have reported on recruitment strategies, or compared strategies, to improve intervention reach have been sparse and what research does exist in this area has been limited (7, 8). In some cases, research has focused on potential participant enablers and barriers to participation (9). Other studies have examined recruitment only from the perspective of providers or physician referrals (10). Still others have examined recruitment relative to a single intervention structure (e.g., group-based) (11) or health behavior outcome (e.g., physical activity) (12).

When considering the reach of health behavior interventions, there are several factors that are hypothesized to determine success (13). These factors include the characteristics of the (1) intended audience, (2) delivery setting and staff, (3) intervention, (4) external factors, and the (5) strategies used to recruit participants (14, 15). Addressing each of these factors within a single study is impractical and the ability to examine potential interactions is likely only possible through a review of literature that has examined reach across a number of populations, delivery settings, intervention structures and foci, and recruitment strategies.

In addition, understanding the underlying mechanisms by which strategies to improve reach achieve a high and representative number and proportion of participants from the intended audience will advance scientific understanding and provide practical principles that can be used to develop additional successful strategies (16). The Practical, Robust, Implementation, and Sustainability Model and Reach, Effectiveness, Adoption, Implementation, and Maintenance Framework (PRISM/RE-AIM) both provide useful guidance on identifying potential mechanisms and ways to operationalize intervention reach. Specifically, the RE-AIM Framework is one of the few dissemination and implementation science frameworks that provides direction on how best to operationalize reach with an emphasis on the number, proportion, and representativeness of participants who are exposed to a recruitment strategy, engage in the recruitment process, and are enrolled in an evidence-based intervention (4). PRISM provides a set of explanatory constructs that can act as contextual moderators or mechanistic mediators in the success of recruitment strategies intended to improve reach which include (1) the multi-level/multi-sector perceptions of a given intervention, (2) multi-leveled staff and setting characteristics, (3) the implementation and sustainability infrastructure of intervention delivery sites, and (4) external environmental factors (15). These constructs provide an opportunity to generate hypotheses to improve dissemination and implementation outcomes that can be used to characterize potential mechanisms (17). Of specific relevance to understanding the utility of strategies to improve reach, PRISM/RE-AIM includes hypotheses related to participant, delivery staff, organizational perceptions and characteristics, the implementation and sustainability infrastructure, characteristics of the intervention, and external factors that may mediate or moderate success (15). The primary objective of this review is to identify recruitment strategies to improve the reach (defined as number, proportion, and representativeness of eligible people) of lifestyle interventions focused on health promotion, disease prevention, and self-management for children and adults delivered in community or clinical settings.

## Methods

We conducted a scoping review following the *JBI Manual for Evidence Synthesis* guidance (18). Using the framework as outlined by *Arksey and O’Malley,* we organized our scoping review into five stages: (1) identifying the research question; (2) identifying relevant studies; (3) selecting the studies; (4) charting the data; and (5) collating, summarizing and reporting the results (19). As the *JBI Manual for Evidence Synthesis* states, “scoping reviews can be used to map the key concepts that underpin a field of research… the three most common reasons for conducting a scoping review [are] to explore the breadth or extent of the literature, map and summarize the evidence, and inform future research.” (20) For transparency and reproducibility, we adhered to the Preferred Reporting Items for Systematic Reviews and Meta-Analyses extension for scoping reviews (PRISMA-ScR) (21) and searches (PRISMA-S) (22) for reporting our literature search and review results. The protocol was registered on the Open Science Framework (osf.io) and is available at https://doi.org/10.17605/OSF.IO/3G68B. See Appendix E for differences between the protocol and manuscript.

### Identifying the research questions

We used JBI’s mnemonic Population-Concept-Context (PCC) framework to frame our research question and the eligibility criteria (18). Our main research question is “What is known about the use of different types of recruitment strategies to improve the reach of evidence-based lifestyle interventions and how they are reported?” We broadly defined lifestyle interventions to include those aimed at health promotion, disease prevention, and self-management. Further, we were interested in categorizing and comparing strategies based on recruitment success operationalized as participation rate and representativeness. Our secondary purpose was to understand the underlying mechanisms by which strategies improve reach. Here, we addressed two additional questions: (1) to what degree does the (a) intended audience, (b) delivery setting and staff, (c) intervention characteristics, and (d) external factors influence the success of different recruitment strategies? And (2) what are the underlying mechanisms by which successful strategies achieve high reach?

### Identifying relevant studies

An information specialist (MMM) developed the search strategies using a combination of keywords and database subject headings for the primary databases (Medline) from sentinel studies (i.e., studies identified at protocol stage that examined the utility of recruitment strategies) and team feedback; a librarian (TC) then translated the strategy to the other selected databases. Library colleagues (AM) peer reviewed the strategy according to Peer Review of Electronic Search Strategies (PRESS) guidelines, a structured process to “identify search errors and improve the selection” of controlled vocabulary headings and keyword terms to “enhance the quality and comprehensiveness of the search” which populates the evidence base for the review (23). Databases included Medline (Ovid), Embase (embase.com), CINAHL Complete (Ebscohost), APA PsycINFO (Ebscohost), and Dissertation and Theses Global (ProQuest). The database results were retrieved on March 2–3, 2023. No date limits or other filters, such as language or publication type, were applied. Citation management and duplication detection and removal were accomplished with EndNote, version 21 (Clarivate). No grey literature (i.e., non-commercial publications from government, business, professional organization, or conferences) was searched (24). For studies meeting the inclusion criteria, references were also evaluated for relevancy and potential inclusion. Detailed search strategies are included in Appendix A. The PRISMA-ScR and PRISMA-S Checklists are in Appendix B.

### Eligibility criteria

Our inclusion criteria (PCC) (18) defined participants as children or adults of any age, gender, race, or ethnicity. We further defined inclusion based on our overall concept as studies with (a) at least one recruitment strategy for a lifestyle intervention, (b) information on the recruitment strategy protocol, (c) data on number of people recruited and number of people exposed to recruitment efforts, and (d) a focus on lifestyle intervention targeting physical activity, dietary intake, weight loss, weight loss maintenance, obesity prevention, diabetes prevention, or diabetes self-management. Finally, from the perspective of context we included studies in community or clinical settings from around the world that used experimental or quasi-experimental (including single group observational) designs.

Exclusion criteria around *concept* included studies in which the (a) recruitment strategy is not specified, (b) no reach outcomes are reported, or (c) recruitment is for non-lifestyle interventions, such as pharmaceutical trials; and *study design* exclusion criteria involved cross-sectional evaluation of a single recruitment strategy. Non-English studies would be excluded at full-text review.

### Study selection

We used Covidence (Veritas Health Innovation), an online systematic reviewing platform to screen and select studies. Two reviewers from a pool of six (EM; EP; JH; LH; MM; PE) independently screened titles and abstracts, then two reviewers from a pool of eight (BG; EP; JC; LH; MM; PE; SS; TM) independently reviewed the full text for inclusion based on our eligibility criteria. When no consensus could be reached between the two reviewers, a third reviewer (BG; EP; JC; LH; MM; PE; SS; TM) was the deciding vote. No artificial intelligence (AI) tools were used in the conduct of the review, although the team did screen all studies using the ‘most relevant’ sort option in Covidence, which uses machine learning (active learning) to show studies by predicted relevance.

### Data charting

Prior to finalization of the protocol, two reviewers (MM; PE) piloted our data charting form using sentinel articles. Data was charted from our included studies by two reviewers (EP; JC; LH; MB; MM) using Microsoft Excel. A third reviewer (EP; JC; LH; MB; MM) who was not involved in the data charting merged the data from the initial two reviewers. Data elements included year of publication, methods, recruitment strategy, lifestyle intervention type, comparison conditions (if relevant), and PRISM/RE-AIM factors related to recruitment strategy implementation and outcomes.

In compliance with scoping review methodology, no quality assessment of included studies was conducted, as our goal was to rapidly map the literature.

## Results

**Figure 1:**
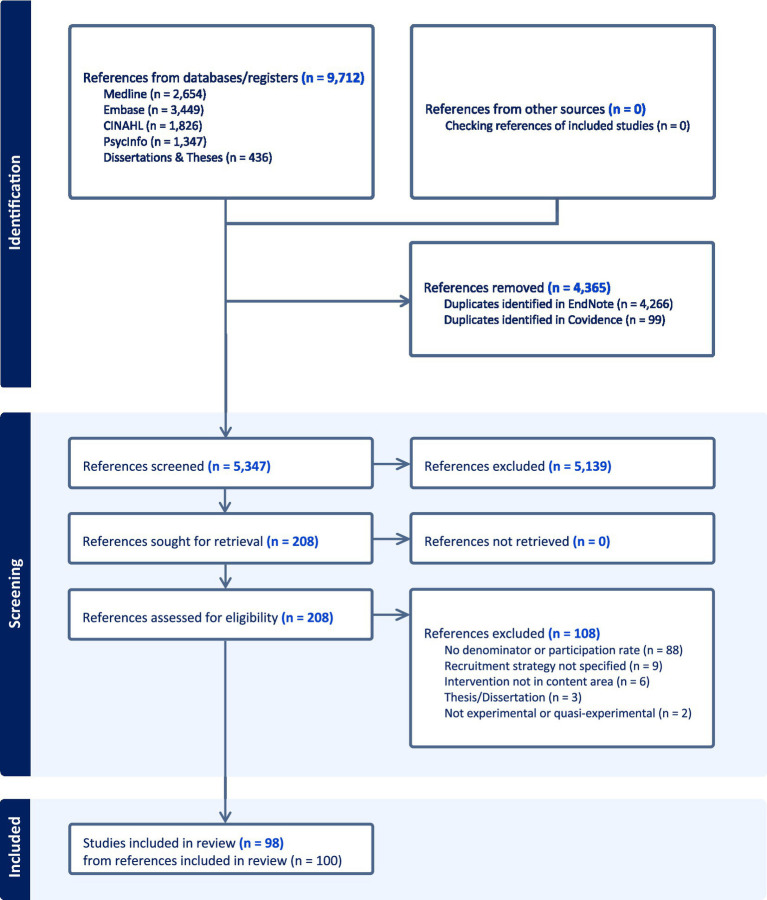
PRISMA flowchart of identified, screened, and included studies.

We identified 9,712 references from our database search strategies. After removal of duplicates, 5,347 references were screened at title/abstract, then 208 references were assessed for eligibility through full-text review, and 98 studies from 100 references met our inclusion criteria (25–122). See Figure 1 for our PRISMA flowchart. No relevant studies were identified from checking the references of our included studies. Appendix C is a bibliography of our included studies. Appendix D is a bibliography of our excluded studies with reasons from the full-text screening.

### The type, use, and reporting of recruitment strategies

Table 1 provides the recruitment strategies, participation rate, number of participants, and the focus on the intervention to which participants are being recruited. Table 2 defines the 15 unique reach strategies we identified from the data with an example for each that was derived from the reviewed studies. Of note, there was not consistent labeling of recruitment strategy type across studies and the 15 strategies identified in Table 2 were derived to assist in categorization and review of strategy success. It is noteworthy that we identified strategies that included single approaches (e.g., mass media), blended approaches (e.g., population health management), and combinations of approaches (e.g., community led recruitment). Table 3 presents the study and participant characteristics of the included studies. The geographical locations of the eligible studies were primarily in the United States (*n* = 58) or Europe (*n* = 32). The vast majority of the studies reported observational data relative to the recruitment strategies being described (*n* = 90) with only eight (26, 54, 60, 70, 86, 87, 103, 119) testing across recruitment strategies using a randomized controlled trial. Nearly half of the included articles provided additional references that further characterized the recruitment strategies (*n* = 46), and the evaluation of the recruitment strategy was most often embedded in implementation, effectiveness, or evaluation studies of lifestyle interventions. Most studies also analyzed data at the individual level (*n* = 85), with quantitative methods employed for the majority of the data analysis (*n* = 84). Table 3 also highlights the degree to which the included studies reported on participant characteristics. Nearly all included studies defined the population intended to benefit from the study (*n* = 95), including the age range (*n* = 78). However, specific information related to the gender [*n* = 17 (25, 26, 31, 32, 35, 42, 43, 65, 66, 71, 76, 85, 90, 105, 106, 110, 120)], race, and ethnicity [*n* = 14 (25, 26, 33, 45, 46, 71, 80, 92, 93, 98, 106, 112, 120, 122)] of the intended population was reported much less frequently. Finally, study goals for representation across sub-groups in the population was only reported in about one third of studies [*n* = 29 (25, 26, 29–31, 33, 34, 44, 45, 50, 51, 54, 64, 67, 69–72, 78, 79, 88, 91, 94–96, 102, 111, 119, 120)].

**Table 1:** Overview of recruitment strategies, participation rate, number of participants and lifestyle intervention type of the included studies in alphabetical by author.

Study	Recruitment strategies used	Reach-participation rate of primary strategy (*n*, participants)	Lifestyle interventions
Adams 2016 (25)	Bundled—Direct Mail, Orientation Events, Point of Care Referrals	11.3% (248)	Self-management
Alexander 2008 (26)	Financial Incentives	4.3% (531)	Health promotion
Bajraktari 2022 (27)	Bundled—Direct Mail, Flyers, Mass Media, Place-Based, Social Media	4.7% (173)	Health promotion
Bayley 2018 (28)	Population Health Management	16.7% (1489)	Health promotion
Bean 2021 (29)	Bundled—Flyers, Place-Based Strategies, Population Health Management, Social Media	12% (271)	Disease prevention
Befort 2020 (30)	Bundled—Point of Care Referrals, Population Health Management, Print Advertising, Social Media	15.7% (1432)	Self-management
Benedetti 2020 (31)	Bundled—Place-Based Strategies, Point of Care Referrals, Flyers, Mass media	11.5% (114)	Health promotion
Bracken 2019 (32)	Mass Media	5% (418)	Health promotion
Brewer 2018 (33)	Bundled—Place-Based Strategies, Flyers, Mass Media, Print Advertising	17% (51)	Health promotion
Brierley 2022 (34)	Bundled—Direct Mail, Place-Based Strategies, Snowball recruitment, Social Media	12% (24)	Health promotion
Brown 2012 (35)	Direct Mail	1% (121)	Self-management
Brunisholz 2017 (36)	Point of Care Referral	8.4% (573)	Disease prevention
Carter 2015 (37)	Point of Care Referral	33% (72)	Health promotion
Chinn 2006 (38)	Point of Care Referral	42% (353)	Health promotion
Chow 2020 (39)	Bundled—Direct Mail, Financial Incentives	46.1% (342)	Health promotion
Clark 2018 (40)	Place-Based	46.8% (1709)	Health promotion
Coughlin 2022 (41)	Population Health Management	1.4% (1021)	Disease prevention
Crane 2016 (42)	Direct Mail	1.3% (807)	Disease prevention
Daley 2008 (43)	Population Health Management	23.1% (28)	Health promotion
Dettlaff-Dunowska 2022 (44)	Point of Care Referral	89.4% (152)	Self-management
Eakin 2007 (45)	Bundled—Flyers, Mass Media, Population Health	33% (200)	Health promotion
Effoe 2016 (46)	Population Health Management	22.4% (160)	Health promotion
Estabrooks 2008 (47)	Bundled—Community-led, Flyers, Mass Media, Print Advertising	1% (5991)	Health promotion
Felix 2012 (48)	Bundled—Flyers, Orientation Events, Place-Based Strategies	27.9% (228)	Health promotion
Franklin 2006 (49)	Bundled—Direct Mail, Flyers, Orientation Events, Place-Based Strategies	24% (1106)	Health promotion
Garip 2017 (50)	Bundled—Place-based, Point of Care, Print Advertising	6% (58)	Health promotion
Ghai 2014 (51)	Population Health Management	7.6% (361)	Health promotion
Glasgow 2000 (52)	Population Health Management	76% (320)	Health promotion
Glasgow 2007 (53)	Population Health Management	5% (909)	Health promotion
Gopalan 2016 (54)	Direct Mail	13% (462)	Health promotion
Guertler 2017 (55)	Place-Based Strategies	56% (376)	Disease prevention
Harden 2014 (56)	Bundled—Direct mail, Flyers, Mass Media, Print Advertising, Snowball	0.3% (307)	Disease prevention
Hirsch 1992 (57)	Population Health Management	58% (2512)	Health promotion
Horowitz 2009 (58)	Bundled—Community Led Recruitment, Orientation Events, Point of Care Referrals	18% (99)	Disease prevention
Jago 2019 (59)	Place-based Strategies	43% (459)	Health promotion
Jalkanen 2021 (60)	Bundled—Mass Media, Place-Based Strategies, Social Media	86% (5882)	Disease prevention
Johnson 2022 (61)	Point of Care Referral	25% (82)	Health promotion
Jong 2020 (62)	Place Based Strategies	53.9% (1543)	Disease prevention
Kerry 2018 (63)	Direct Mail	11% (1150)	Health promotion
Kirley 2021 (64)	Population Health Management	2.4% (116)	Health promotion
Kozica 2015 (65)	Bundled-Direct Mail, Place-Based, Flyers, Mass Media, Print Advertising, Social Media	6% (649)	Health promotion
Lawlor 2019 (66)	Place Based Strategies	87% (40)	Health promotion
Lewis 2017 (67)	Point of Care Referral	24.7% (40)	Health promotion
Linnan 2002 (68)	Place Based Strategies	55% (1906)	Disease prevention
Linnan 2012 (69)	Bundled—Direct mail, Flyers, Mass Media, Social Media	44% (1004)	Health promotion
Liu 2014 (70)	Bundled—Direct mail, nonfinancial incentives	53% (38835)	Self-management
Liu 2020 (71)	Bundled —place based, flyers, social media	14% (228)	Health promotion
Long 2010 (72)	Financial Incentives	69.7% (3069)	Health promotion
Madsen 2014 (73)	Point of Care Referral	27% (35)	Health promotion
Markert 2013 (74)	Population Health Management	9% (303)	Disease prevention
Mas-Alos 2021 (75)	Point of Care Referral	1.2% (178)	Self-management
McEachan 2016 (76)	Place-based strategies	30% (120)	Disease prevention
Mills 1996 (77)	Bundled—Direct Mail, Flyers, Orientation Events, Telephone Outreach	39.6% (227)	Disease prevention
Mullane 2019 (78)	Bundled—Direct Mail, Place-Based Strategies, Flyers, Orientation Events, Telephone Outreach	48% (632)	Health promotion
Oddone 2018 (79)	Population Health Management	13.9% (417)	Health promotion
Okhomina 2020 (80)	Bundled —Direct Mail, Flyers, Orientation Events	8.1% (375)	Disease prevention
Olij 2019 (81)	Bundled —Place-Based Strategies, Flyers, Mass Media	0.4% (450)	Health promotion
Parkinson 2020 (82)	Point of Care Referral	21% (2195)	Health promotion
Parra-Medina 2004 (83)	Population Health Management	17.1% (189)	Health promotion
Partridge 2015 (84)	Social Media	21.2% (250)	Health promotion
Peck 2008 (85)	Bundled—Place-Based Strategies, Flyers, Mass Media, Orientation Events, Print Advertising, Snowball, Social Media	62.2% (430)	Health promotion
Peels 2012 (86)	Direct Mail	14.2% (1729)	Health promotion
Porter 2021 (87)	Point of Care	8.8% (40)	Health promotion
Porter 2021 (87)	Population Health Management	10.8% (58)	Health promotion
Ramsay 2020 (88)	Bundled—Engage Leaders, Place-Based Strategies, Population Health Management, Orientation Events, Print Advertising, Snowball Recruiting	37% (453)	Disease prevention
Robroek 2012 (89)	Place-Based Strategies	7.2% (924)	Health promotion
Samuel-Hodge 2012 (90)	Bundled—Direct Mail, Flyers, Mass Media, Print Advertising	44% (189)	Health promotion
Sanchez 2016 (91)	Point of Care Referral	0.7% (454)	Disease prevention
Santoyo-Olsson 2011 (92)	Bundled—Community-Led, Mass Media, Orientation Events, Snowball	42.8% (238)	Health promotion
Sharpe 2021 (93)	Bundled-Direct Mail, Engaged Leaders, Mass Media, Place-Based Strategies	8.8% (527)	Self-management
Snyder 2009 (94)	Population Health Management	3.2% (641)	Disease prevention
Speck 2010 (95)	Direct Mail	47% (619)	Health promotion
Spittaels 2007 (96)	Flyers	13.4% (171)	Health promotion
Stevens 2008 (97)	Bundled—Direct Mail, Place-Based Strategies, Snowball	12.3% (351)	Health promotion
Stineman 2011 (98)	Population Health Management	31.8% (204)	Disease prevention
Stopponi 2009 (99)	Bundled—Direct Mail, Financial Incentives	8.9% (2540)	Health promotion
Taradash 2015 (100)	Bundled—Direct Mail, Engage Leaders, Flyers, Place-Based Strategies	50.6% (89)	Health promotion
Tercyak 2006 (101)	Bundled —Direct Mail, Flyers	31% (75)	Health promotion
Terry 2010 (102)	Direct Mail	39% (631)	Disease prevention
Thilsing 2021 (103)	Direct Mail	46.9% (2171)	Disease prevention and self-management
Tidwell 2004 (104)	Bundled—Direct Mail, Telephone Outreach	28% (504)	Health promotion
Toobert 2002 (105)	Bundled-Point of Care Referrals, Population Health management	14.9% (76)	Health promotion
Turner 2021 (106)	Population Health Management	44% (290)	Disease prevention
vanderGiesen 2010 (107)	Point of Care Referral	2% (150)	Disease prevention
vanDongen 2016 (108)	Population Health Management	54% (316)	Health promotion
vanHolland 2017 (109)	Opt Out Enrollment	84% (220)	Health promotion
Verburgh 2022 (110)	Bundled—Direct Mail, Flyers, Place-Based Strategies, Snowball	17% (70)	Health promotion
Vermunt 2010 (111)	Population Health Management	5.8% (925)	Disease prevention
Vincent 2013 (112)	Bundled—Flyers, Place-Based Strategies, Point of Care Referrals, Snowball	20.8% (58)	Health promotion
Wages 2010 (113)	Community-Led Recruitment	2% (19281)	Health promotion
Ward 2016 (114)	Point of Care Referral	(17%) 166	Disease prevention
Ware 2008 (115)	Flyers	11.5% (265)	Health promotion
Weston 2021 (116)	Place-Based Strategies	37.3% (41)	Self-management
Wilson 2021 (117)	Population Health Management	5.8% (599)	Health promotion
Withall 2012 (118)	Bundled—Community-Led, Flyers, Snowball, Social Media	10.2% (364)	Disease prevention
Xiao 2015 (119)	Population Health Management	1.9% (199)	Disease prevention
Yancey 2001 (120)	Bundled—Direct Mail, Flyers, Mass Media, Snowball, Social Media	18.8% (893)	Disease prevention
Yank 2013 (121)	Population Health Management	44% (241)	Self-management
Yeary 2019 (122)	Bundled—Community-Led, Flyers, Place-Based	84% (437)	Self-management

**Table 2:** Reach strategies, definitions, and examples.

Strategy	Definition	Examples
Direct mail	Recruitment resources are sent via mail, email, or text to a known list of people that are at high likelihood to be part of the intended audience	Young-adult households randomly assigned to receive recruitment information in the mail (42)
Community led recruitment	Community members or organizations develop and implement locally relevant recruitment activities	Local volunteer task force developed and distributed of promotional materials designed specifically for the program (113)
Engage leaders to support recruitment	Invite local community or organizational leadership to support or engage in recruitment strategies	Local community leader names included on recruitment materials (93).
Financial incentives	Monetary renumeration for recruitment itself or intended to increase recruitment	Different groups were offered $25 or $50 checks at recruitment (39)
Flyers, posters, brochures	Brief, stand alone, written documents with program information	Posters and flyer advertisements placed in the clinic (115)
Mass media	Recruitment information is shared via television/radio	Local media advertisements and public service announcements (56)
Non-financial incentives	Participants receive rewards or prizes for recruitment, retention, or engagement	Participants received points for enrollment and engagement with program components. The points could be cashed in for branded materials (e.g., mugs) (70)
Orientation events	Sessions provided for potential participants that are located at the program or study location	Recruitment is completed at kickoff or orientation events at the study or intervention site (50)
Place-based strategies	Recruiters physically attend locations where the intended audience aggregates	Information booths in the workplace or recruitment of the intended audience through home visits associated with existing service provision (34, 97)
Point of care referrals	Referrals by healthcare providers during medical appointments	Physician toolkit with a laminated pocket reference, program information, and referral cards to be used during a routine appointment (61)
Population health management	Electronic health record review with direct outreach to potentially eligible participants using mail, email, text, or a patient portal	Lists of potential participants identified using the electronic health record, then reviewed by their physician for approval. Those approved received an invitation letter from their physician with an opt out card & info for enrollment (117)
Print advertising	Information is provided in newspapers, local magazines, or organizational announcements	Recruitment information was included in a local newspaper advertisement (65)
Snowball	Participants to invite friends, family, and co-workers	Participating employees were encouraged to invite coworkers (34)
Social media advertising	Posting or sharing information on the internet or through social media platforms	Online presentations and posts using social networks platforms (e.g., Facebook) (27)
Telephone outreach	Calls or texts to encourage participation	Telephone or text outreach to a list of potential participants (104)

**Table 3:** Study and participant characteristics.

Data element (*n* = 98)	Description	Reported—*n* (%)
Study characteristics		
Study location	United States	58 (59%)
	(25, 26, 30, 33, 35, 36, 39, 41, 42, 45–49, 51–53, 56–58, 61, 64, 67–69, 71–73, 77–80, 82, 83, 85, 87, 88, 90, 92–95, 98–102, 104–106, 112–114, 117, 119–122)	
	Canada	3 (3%)
	(29, 40, 70)	
	Europe	32 (33%)
	(27, 28, 34, 37, 38, 43, 44, 50, 55, 59, 60, 62, 63, 66, 74–76, 81, 86, 89, 91, 96, 97, 103, 107–111, 115, 116, 118)	
	Africa	1 (1%)
	(54)	
	Central and South America	1 (1%)
	(31)	
	Asia	0 (0%)
	Australia/New Zealand	3 (3%)
	(32, 65, 84)	
Study conducted in low-middle income country	Yes	1 (1%)
	(31)	
	No	97 (99%)
Purpose context	Planning and development	0 (0%)
	Implementation of recruitment strategy	69 (70%)
	(25, 27, 30, 33, 35, 39, 41–43, 45–51, 53–61, 63–71, 73–80, 83–85, 87–90, 92, 94, 97, 99, 100, 102–106, 110–112, 114, 115, 118–122)	
	Evaluation of recruitment strategy	28 (29%)
	(28, 29, 31, 32, 34, 36–38, 40, 44, 52, 62, 72, 81, 82, 86, 91, 93, 95, 96, 98, 101, 107–109, 113, 116, 117)	
	Dissemination	1 (1%)
	(26)	
	Sustainment	0 (0%)
	Other	0 (0%)
Study design used to evaluate recruitment strategy	RCT	8 (8%)
	(26, 54, 60, 71, 86, 87, 103, 119)	
	Observational	90 (92%)
	(25, 27–53, 55–59, 61–69, 71–85, 88–102, 104–118, 120–122)	
Companion article	Was there a companion article to this intervention?	
	Yes	46 (47%)
	(28, 30, 32, 33, 35, 37, 38, 40–46, 49, 53, 60, 62, 63, 65, 68, 69, 75, 76, 79, 84, 86–90, 96–99, 103, 106, 108–110, 114–117, 121, 122)	
	No	52 (53%)
	(25–27, 29, 31, 34, 36, 39, 47, 48, 50–52, 54–59, 61, 64, 66, 67, 70–74, 77, 78, 80–83, 85, 91–95, 100–102, 104, 105, 107, 111–113, 118–120)	
Level/unit of analysis	What level is the study randomizing at?	
	Individual (patient/participant)	85 (87%)
	(25, 26, 28–31, 33, 35–52, 54, 55, 57–66, 68–82, 84–91, 93–103, 105, 107, 109, 111–121)	
	Recipient (provider/implementation staff)	3 (3%)
	(34, 53, 108)	
	Setting	3 (3%)
	(56, 83, 110)	
	Community	3 (3%)
	(27, 106, 122)	
	Multi-level (nested RCT)	2 (2%)
	(32, 104)	
	Other	2 (2%)
	(67, 92)	
Methods used:	Quantitative	84 (86%)
	(25–32, 34, 35, 37, 38, 40, 42–62, 64, 66, 67, 72–78, 80–82, 84–113, 115–117, 119–122)	
	Mixed methods (only if quant/qual is integrated)	14 (14%)
	(33, 36, 39, 41, 63, 65, 68–71, 79, 83, 114, 118)	
Lessons learned (positive and negative)	Briefly described the lessons learned in 2–3 sentences.	
	Yes	13 (13%)
	(26, 40, 52, 58, 66, 75, 82, 84, 87, 90, 95, 104, 122)	
	No	85 (87%)
	(25, 27–39, 41–51, 53–57, 59–65, 67–74, 76–81, 83, 85, 86, 88, 89, 91–94, 96–103, 105–121)	
Participants		
Intended population description	Description of the population that is intended to benefit from a lifestyle intervention.	95 (97%)
	(25–37, 39–112, 114–117, 119–122)	
Intended population age range	Defined age range described in manuscript	78 (80%)
	(25–38, 40–48, 50–57, 59, 60, 62–71, 73–78, 81, 83–86, 88, 90–94, 96–101, 103–105, 108, 110–112, 116, 117, 119, 120, 122)	
Intended population gender	As defined in the manuscript.	17 (17%)
	(25, 26, 31, 32, 35, 42, 43, 65, 66, 71, 76, 85, 90, 105, 106, 110, 120)	
Intended population race and ethnicity	As defined in the manuscript.	14 (14%)
	(25, 26, 33, 45, 46, 71, 80, 92, 93, 98, 106, 112, 120, 122)	
Representativeness of enrolled participants	The degree to which the enrolled sample is representative of the defined intended population.	29 (30%)
	(25, 26, 29–31, 33, 34, 44, 45, 50, 51, 54, 64, 67, 69–72, 78, 79, 88, 91, 94–96, 102, 111, 119, 120)	

Table 4 presents concept characteristics of the reach strategy and lifestyle intervention of our included studies. The studies describe the reach strategy and lifestyle interventions but were inconsistent in reporting all factors. About half of the studies (*n* = 49) reported if the goal of the recruitment strategy was to improve either or all reach outcomes, such as the number of participants enrolled, the participation rate, or representativeness of populations experiencing disparities. Areas of relatively high reporting of the reach strategy components included the setting (*n* = 93), the channel of delivery (*n* = 93), and the staff involved in implementing the strategy (*n* = 73). Specifically, of the studies reporting lifestyle intervention delivery setting, community (20%), healthcare (15%), and home-based (14%) were the most frequently described. Remote or online interventions were also reported for 16% of the studies. Areas with low reporting included the cost of strategy [*n* = 13 (25, 33, 41, 46, 48, 53, 80, 93, 94, 100, 103, 115, 119)], a guiding theory for the strategy [*n* = 7 (41, 44, 48, 62, 68, 72, 105)], and the intended mechanism of action [*n* = 5 (25, 37, 50, 53, 85)]. When considering lifestyle intervention contextual factors, the included studies reported the intervention setting (*n* = 76), delivery channel (*n* = 68), and format (*n* = 62). Information on intervention implementation staff and number (*n* = 52), participant contact (*n* = 50), timing (*n* = 48), and duration of sessions (*n* = 29) were less reported.

**Table 4:** Reporting of concept characteristics of the reach strategies and the lifestyle interventions.

	Description	Reported—*n* (%)
Concept-reach strategy		
Reach outcome targeted	Number enrolled, participation rate, representativeness (26, 28, 36, 37, 39, 41–43, 48–51, 57, 58, 60, 62, 64, 65, 67, 69, 72, 74, 75, 77, 79, 81, 84, 85, 88–90, 92, 94, 97–99, 101, 104, 106–109, 111, 113, 117, 118, 120)	49 (52%)
Who implemented the reach strategy	Description of the staff/organization responsible for implementing the strategy (25–27, 29–32, 34, 36–40, 44, 45, 47–53, 55, 58–61, 64, 66–71, 73–77, 79–83, 85, 86, 88, 89, 92–94, 96–99, 101–104, 106–108, 110–115, 117, 119–122)	73 (75%)
Reach strategy setting	Where the reach strategy is implemented such as community, school, faith-based, workplace, health department, national health initiative, clinic, etc. (25–34, 36–75, 77–86, 88–105, 107–120, 122)	93 (95%)
Reach strategy delivery channel	Describe how the reach strategy was delivered in terms of channel—in-person, telephone, smartphone app, internet, etc. (25–37, 39–42, 44–52, 54–108, 110–113, 115–122)	93 (95%)
Temporality of strategy	Description of when the strategy is implemented (25, 27, 30, 31, 34, 38, 39, 41, 42, 46–48, 50–54, 56, 57, 60–62, 64, 65, 68, 69, 71, 77, 80, 82, 85, 86, 88, 90–92, 94, 97–100, 102, 103, 106–108, 110–115, 117, 119, 122)	55 (56%)
Dose	Description of the number and duration of strategy contacts (31, 36, 37, 39, 41, 44–50, 52–55, 58, 62, 64, 68–70, 73–76, 80, 82, 84–87, 91–94, 96, 98–100, 103–106, 108, 110, 111, 115, 117, 121)	50 (51%)
Cost of reach strategy	Description of cost of recruitment strategy (25, 33, 41, 46, 48, 53, 80, 93, 94, 100, 103, 115, 119)	13 (14%)
Intended mechanism of action	Specific constructs that mediated the relationship between the strategy and reach outcomes—deductive analysis based on PRISM/RE-AIM constructs (25, 37, 50, 52, 85)	5 (5%)
Identified guiding theory	Specific reference to a guiding theory, model, or framework (41, 44, 48, 62, 68, 72, 105)	7 (7%)
Context-lifestyle intervention		
Lifestyle intervention setting	Describe where the lifestyle intervention takes place—community, school, faith-based organization, workplace, clinic, etc. (25–29, 31–35, 38, 42–54, 57–63, 65–72, 74, 77, 78, 80–90, 92, 94–99, 101–103, 105, 107, 109–112, 114–116, 118–120, 122)	76 (78%)
Lifestyle intervention implementation staff	Description of those responsible for implementing the lifestyle intervention including level of expertise (26, 27, 29–31, 35, 38, 40, 42, 44, 49, 54, 58, 59, 61, 63, 65–70, 72, 75, 76, 78, 81–83, 85, 87–90, 92–94, 96–98, 101, 102, 109–114, 118–120, 122)	52 (53%)
Lifestyle intervention delivery channel	Describe how the lifestyle intervention is delivered in terms of channel—in-person, telephone, smartphone app, internet, etc. (25, 26, 28–34, 36, 38, 40, 43–46, 48–50, 52–54, 56–63, 66–72, 74, 77–79, 83–97, 102, 104, 105, 109–111, 113, 116, 118–120, 122)	68 (69%)
Lifestyle intervention format	Describe the format of lifestyle intervention delivery in terms of individual versus group (25–31, 33–35, 40, 42–45, 48, 49, 52–54, 56, 57, 59–63, 65, 67, 68, 70, 72, 75, 76, 78, 79, 82, 84, 85, 87–89, 92–98, 102, 104, 105, 109–112, 114, 116, 118–120, 122)	62 (63%)
Number of lifestyle intervention of contacts	Total number of encounters with participants. Could include face-to-face meetings, telephone calls, newsletters etc. (26, 28, 29, 35, 40, 44, 48, 49, 52–61, 63, 65, 67, 68, 70, 74–79, 82, 83, 85, 87, 88, 92–94, 97, 102, 104, 105, 109–111, 114, 118–120, 122)	50 (51%)
Timing of lifestyle intervention contacts	Describe when the intervention contacts occur over the course of the intervention (26, 28–30, 34, 40, 44, 45, 48, 49, 52, 53, 55, 57–61, 63, 65, 67–70, 74–76, 78, 81–83, 85, 87, 88, 92–94, 97, 104, 105, 110, 111, 114, 116, 118–120, 122)	48 (49%)
Duration of lifestyle intervention contacts	Length of each intervention contact (28, 31, 40, 44, 46, 48, 49, 55, 59, 60, 67, 70, 74–77, 79, 85, 88, 90, 92–94, 97, 104, 111, 114, 118, 119)	29 (30%)

### The success of recruitment strategies

Across the included studies, the average number of recruitment strategies applied was 2.5 (± 2.1; see Table 1). Nearly half (40/98) of the studies included bundled strategies and did not differentiate reach based by single strategies. These bundled strategies averaged a participation rate of 16% of the intended population. Strategies consisting of population health management [*n* = 21 (28, 41, 43, 46, 51–53, 57, 64, 74, 79, 83, 87, 94, 98, 106, 108, 111, 117, 119, 121)], point of care referrals [*n* = 13 (36–38, 44, 61, 67, 73, 75, 82, 87, 91, 107, 114)], place-based strategies [*n* = 9 (40, 55, 59, 62, 66, 69, 76, 89, 116)], and direct mail [*n* = 8 (35, 42, 54, 63, 86, 95, 102, 103)] reported, respectively, median participation rates of 12, 21, 43, and 13%. Eight studies that focused on Black/African American participants reported a 17% participation rate (25, 33, 80, 93, 98, 106, 120, 122) relative to a 21% participation rate when racial groups were not differentiated. Similarly, only 17 studies differentiated on gender, with three studies focusing on men reporting an 8% participation rate (32, 42, 106) relative to a 22% participation rate for women. Studies did report on ages and the reported participation rates were highest for children (43%) and lowest for older adults (16%). Participation rates based on delivery channel were clustered between 19 and 22% except for word of mouth which reported 9% participation rate across six studies (26, 48, 62, 88, 101, 117). Further, differences in who delivered the recruitment strategy, study design, and intervention type did not appear to result in differences in reported participation rate, ranging from 18 to 25% of the intended population.

#### What is known about theoretical approaches and mechanisms of improved reach?

Twenty-four included studies described using some portion of the PRISM/RE-AIM framework in the design, implementation, or evaluation of their study (25–27, 33, 36, 38, 40, 44, 51, 54, 59, 60, 65, 72, 78, 85, 88–90, 110, 112, 118, 120, 122). However, only seven studies reported a theoretical framework that guided recruitment strategy development [e.g., Diffusion of Innovation (123), Self-Determination Theory (124), or Social Marketing (125)] (41, 44, 48, 62, 68, 72, 105) and only five highlighted potential mechanisms of improved reach (e.g., confidence or social support) (25, 37, 50, 52, 85). No studies examined differences in participation rates based on the intended audience, delivery setting and staff, intervention characteristics, or external factors—the PRISM/RE-AIM contextual factors were hypothesized to moderate and mediate successful reach.

## Discussion

The objective of this review was to identify recruitment strategies to improve the reach of lifestyle interventions focused on health promotion, disease prevention, and self-management for children and adults delivered in community or clinical settings. We also intended to report on the application of theory or conceptual models to improve the reach of lifestyle interventions across participants of all ages. We found that it appears as though place-based strategies achieve the highest participation rate, followed by point of care referrals, bundled strategies, and population health management. Of note, several strategies have only been used as part of recruitment bundles (e.g., engaging leaders to support recruitment or orientation events), making it difficult to determine the utility of these strategies.

In connection with this research topic area focused on aging, we documented that studies that focused on children recruited an average of 43% of the intended audience compared to 25% in adults and 16% for older adults when examined independently or with the full age range of adulthood. Compared to their younger counterparts, older adults may face unique barriers, such as deteriorating health and increasing social isolation, for participating in health promotion, disease prevention, and disease self-management interventions (126). In addition, older adults constitute a greater proportion of the population size in rural communities than urban communities, and older adults in rural communities can have fewer opportunities for such interventions (i.e., unavailability of the interventions) and greater transportation barriers to the interventions that may also influence reach (127).

However, it is likely premature to suggest that children are more likely to engage with lifestyle interventions; it may be more likely that there is a unique context of reach in these different age groups. For example, children and adolescents tend to be physically bound within school settings and have different types of social relationships that can influence recruitment (e.g., peers, teachers, and parents) compared to adults. Likely even more powerful is that recruitment through schools for school-based interventions has an inherently high reach (62). Similarly, a review of participation rates in workplace health promotion programs found an average participation rate just below 50%, which may be less related to the specific strategies and more related to having a known denominator in a setting where lifestyle interventions may be attractive as an employee benefit (128). Another review of enrollment of adults (18 years and older) with cancer and their caregivers in psychosocial or behavioral interventions trials (RCTs) resulted in an average enrollment rate of 33% (129). Both reviews observed participation rates that are higher than our participation rates ranging from 12 to 25% across strategies tested in more than 10 studies. These findings suggest the importance of considering the intended population and setting when planning reach strategies.

Our findings provide several directions for future research that examines the relative utility of different recruitment strategies to improve reach. First, there is a need to better define specific recruitment strategies and improve the application of theory or conceptual models to the design and application of strategies. Second, in addition to providing a recruitment strategy definition, dissemination science as it relates to reach would be better advanced by specifying strategies based on the strategy enactor, components, potential mechanism of improved reach, timing, dose, and intended reach outcome (6). Third, addressing the challenge of improving the reach of lifestyle interventions requires agreement on appropriate assessment of numerators, denominators, and characteristics of potential participants across temporal aspects of reach from exposure to recruitment activities, engagement in the enrollment process, enrollment itself, attendance at intervention sessions, and completion of the intervention (56). Fourth, despite the limitations of the current literature, participant characteristics such as age appear to be related to intervention reach, with higher rates of reach found for younger participants and lower rates with older adults.

Reach is a primary challenge across the spectrum of evidence-based lifestyle interventions whether during efficacy, effectiveness, or implementation trials (56, 87, 130–132). It is unsurprising that we identified 98 unique studies that evaluated or tested different recruitment strategies. We defined 15 unique strategies that were intended to improve intervention reach based on activities described to improve reach across studies (Table 2). However, we acknowledge that these definitions, while helpful, require additional scientific vetting due to potential overlap or muddling of recruitment setting and recruitment strategy. As the Expert Recommendations for Implementation Change compendium of strategies to improve implementation outcomes filled a gap in the implementation science literature (133), future work in this area should focus on addressing the gap in available and consistent labels and definitions for individual-level dissemination strategies that facilitate recruitment of those who would benefit from lifestyle interventions—and allow comparisons across populations, settings, and interventions.

While the field of dissemination and implementation science has a cornucopia of available theories, models, and frameworks, recent scoping reviews have suggested that there is an overemphasis on implementation and an underemphasis on dissemination (134). This may be surprising given the foundational work of Everett Rogers on the Diffusions of Innovation theory, which provides processes and mechanisms for both setting (i.e., adoption of innovations) and individual (i.e., reach of innovations within a population) level dissemination (135, 136). Still, our review supports the conclusion that there has been a lack of reporting the theory applied to the design, testing, and identification of mechanisms of change of strategies intended to improve reach. Indeed, only seven studies (25, 42, 47, 52, 60, 70, 118) referenced a theoretical approach and only five provided a description of potential mechanisms of change (25, 37, 50, 53, 85).

Understanding the underlying mechanisms or reasons why a specific strategy is successful allows for generalizability to other settings and provides guidance for recruitment strategy design in settings with different levels of resources to support recruitment. For example, population health management approaches may be designed to leverage the patient-provider relationship to improve potential participant normative beliefs of the benefits of enrolling in a lifestyle program (i.e., mechanism), which in turn leads to a higher participation rate. If one were to apply concepts from the Theory of Planned Behavior (137) to this example, qualitative and quantitative approaches could be used to see if leveraging the patient-provider relationship resulted in increased perceptions of subjective norm which lead to improved reach. Other authors have also highlighted the importance of understanding the mechanisms by which recruitment strategies can enhance participant engagement and adherence with, and to, evidence-based intervention components (138). Described as adjunctive interventions, Smith and colleagues suggest that methods targeting potential participants for health-focused interventions recruitment strategies can be designed to enhance motivation, self-efficacy, or capacity to engage with a health intervention (138).

In addition to underreporting of underlying theories and mechanisms to improve reach, we found that studies were highly variable in the degree to which they specified strategies based on the way, by who (including demographics and role), how often, and at what dose strategies were applied to improve reach. Important considerations for both the use and success of strategies to improve reach include the cost [reported by only 13 studies (25, 33, 41, 46, 48, 52, 80, 93, 94, 100, 103, 115, 119)], dose [reported by 50 (31, 36, 37, 39, 41, 44–50, 52–55, 58, 62, 64, 68–70, 73–76, 80, 82, 84–87, 91–94, 96, 98–100, 103–106, 108, 110, 111, 115, 117, 121)], and temporality [reported by 55 (25, 27, 30, 31, 34, 38, 39, 41, 42, 46–48, 50–54, 56, 57, 60–62, 64, 65, 68, 69, 71, 77, 80, 82, 85, 86, 88, 90–92, 94, 97–100, 102, 103, 106–108, 110–115, 117, 119, 122)]. Without this information, replication of strategies, and generalizing strategies into typical community or clinical settings is genuinely compromised.

Proctor and colleagues recommend specification of strategies based on the actor (i.e., who enacts the strategy), the action (i.e., the steps involved in the strategy), the factor or people intended to be influenced by the action (i.e., mechanism of change; priority population to be reached), temporality (i.e., when the strategy is used), dose (i.e., the duration of the strategy), the implementation outcome (i.e., fidelity or sustainment), and the justification (i.e., empirical or theoretical rational for using the strategy) (6). Previous reviews of literature using the RE-AIM framework provide direction on specifying factors related to the intended audience, intervention, and delivery settings (7, 139). For example, to improve reporting on characteristics of the intervention into which potential participants are being recruited, we recommend that the target intervention of the recruitment strategy also be specified in terms of the intervention delivery setting, staff, delivery mode and format, number, frequency and length of sessions. Similarly, we recommend defining the intended audience including subgroups that may experience health differences and the plan to assess reach for priority populations including the number, proportion, and representativeness to that priority population (140). In addition, studies that use community-engaged approaches to identify, engage, and enroll participants in lifestyle interventions typically provide details on the co-creation of the lifestyle intervention but do not describe, in detail, the engagement process in the selection and creation of recruitment strategies (23). We recommend that those using participatory approaches to develop recruitment strategies provide detail on the process of partnership development and involvement in recruitment strategy identification, creation, and deployment in addition to specifying the recruitment strategy factors (34).

It is of note that we used the reported participation rates from each included study. Unfortunately, the degree to which studies defined a denominator of those exposed to recruitment strategies varied widely, which makes conclusions around participation rate comparisons challenging. One of the key areas of inconsistency was around the operationalizing and reporting of a specific denominator based on the temporality of the recruitment process. Harden and colleagues highlighted this issue and proposed that proportional reach (i.e., participation rate) should be considered across four indicators (56). These were the proportion of the population (1) exposed to the recruitment strategy, (2) who respond to the recruitment strategy and express interest, (3) who enroll in the intervention, and (4) who attend intervention sessions over time. We would add to this characterization by adding a final temporal state of the proportion who complete the intervention and recommend that representativeness and tracking of priority population rates be assessed across each of these phases.

While our review excluded studies that focused only on recruitment strategies for clinical trials they may provide context to our recommendations given the potential similarities in recruitment barriers and facilitators. For example, our review included surprisingly few studies that applied financial incentives to support recruitment. In clinical trials, financial incentives to address social needs, transportation, and childcare have demonstrated success in recruiting under-represented populations (141). Similarly, strategies that ensure interventions are culturally relevant or have demographic concordance between the recruiter/implementer and participant have also been proposed to improve trust and recruitment into clinical trials (142). These findings may provide alternative methods to categorize strategies based on the underlying barrier or facilitator they are designed to address (e.g., trust, logistics). Still, differences between recruitment for clinical trials and lifestyle change intervention studies likely exist where, in one case, participants are asked to agree to testing a medication or providing biospecimens and, in the other, are asked to enroll in an intervention that promotes healthy behaviors. This raises questions about the characteristic of the innovation and underlying perceptions of the intended study population toward that innovation. These differences likely necessitate the application of different types, dose, and delivery channel of recruitment strategies—excellent areas of future research on strategies to promote lifestyle intervention reach. Finally, even within the variety of lifestyle change interventions it is likely that there are interactions between recruitment strategy type and the format of the intervention.

While this review sheds lights on an important gap in dissemination and implementation science related to strategies intended to improve lifestyle intervention reach, it is not without limitations. We provided labels, definitions, and categories of recruitment strategies for the studies we reviewed, but we anticipate that these labels and definitions are not exhaustive and present opportunities for further refinement. Additionally, although several databases were searched and references of our included studies were reviewed, published or unpublished studies might have been missed due to the vast number of lifestyle intervention studies conducted. We did not reach out to authors to address unreported data or for clarification. Since only eight studies used randomized controlled trials to test recruitment strategy influence on reach with little content overlap between these studies (26, 54, 60, 70, 86, 87, 103, 119), along with no assessment of quality or risk of bias conducted on included studies, the specific results should be interpreted with caution and used as a starting point rather than as a final destination for understanding participation rate. Although active learning was used in Covidence for screening, all studies were screened, selected and extracted in pairs by humans without artificial intelligence (AI) ensuring transparency and reproducibility.

In conclusion, the strengths of our review included a broad conceptualization of recruitment strategies and outcomes across 98 included studies to provide valuable information on available reach strategies, possible definitions, and a resource for others looking for research on improving reach. We analyzed studies to identify effective recruitment strategies for lifestyle interventions aimed at health promotion, disease prevention, and self-management. We found a variety of recruitment strategies, with 32% of studies using multiple strategies. Participation rates ranged from 3 to 41%, with higher rates observed in children (43%) compared to adults (25%) and older adults (16%). Most studies lacked detailed reporting on strategy timing, dose, theoretical basis, and mechanisms of improved reach, making comparisons difficult. The review highlights the need for consistent definitions of eligible participant denominators and the inclusion of theoretical approaches and mechanisms in future studies to enhance the understanding and effectiveness of recruitment strategies. Future research should focus on clearer reporting of recruitment strategies, including timing, dose, and theoretical underpinnings. Identifying and testing mechanisms that improve reach is crucial, as is considering the unique contexts of different age groups and settings. This will help develop more effective recruitment strategies that will improve enrollment for lifestyle interventions across all populations.

## Data Availability

The original contributions presented in the study are included in the article/Supplementary material, further inquiries can be directed to the corresponding author.
