# Supplementary material for: Identifying recruitment strategies to improve the reach of evidence-based health promotion, disease prevention, and disease self-management interventions: a scoping review
Source: Front Public Health. 2025 Apr 1;13:1515042. doi: 10.3389/fpubh.2025.1515042 (PMC12023269; doi:10.3389/fpubh.2025.1515042)
Supplement: Supplementary file 1 [file Supplementary_file_1.docx]

Appendix A: Search Strategies

Databases included Medline (Ovid) 1946-2023, Embase (embase.com) 1974-2023,

CINAHL Complete (Ebscohost) 1937-2023, APA PsycINFO (Ebscohost) 1872-2023, and Dissertation and

Theses Global (ProQuest) 1861-2023. No date limits or other filters were applied.

Medline was searched on September 22, 2022.

Medline was updated and all other database results retrieved on March 2-3, 2023.

# Medline (Ovid)

1 (Recruitment and (PRISM or RE-AIM)).ti,ab,kf,kw. [Recruitment + PRISM/RE-AIM keywords ] 121

2 (Recruitment adj9 reach).ti,ab,kf,kw. [Recruitment + reach keywords ] 493

3 Patient selection/ or (((recruit* or selection*) adj3 (active or criteria or engagement* or method* or outreach or patient* or participant* or passive or reach or "research subject*" or "research volunteer*" or subject or subjects or volunteer or volunteers)) or "selection criteria*" or "selection for treatment*" or "subject selection*").ti,ab,kw,kf. [Recruitment 1 – subject or keyword terms] 270865

4 ((Recruit* or Recruitment) adj6 (active* or passive* or reach or representative or representativeness)).ti,ab,kf,kw. [Recruitment 2 terms] 5384

5 ((enrollment or participation or recruit* or recruitment) adj6 (method* or individuals or patient* or participant* or population or sample or strategy or strategies)).ti,ab,kf,kw. [Recruitment terms 3] 179340

6 or/3-5 [Recruitment set] 357051

7 (Recruitment and reach).ti,ab,kf,kw. [Recruitment + reach ] 2362

8 (enroll* or enrollment*).ti,ab,kf,kw. [Enrollment keyword terms] 449404

9 (reach or outreach).ti,ab,kf,kw. [Reach/outreach keywords] 208957

10 representativeness*.ti,ab,kf,kw. [Representiveness keywords] 4352

11 implementation.ti,ab,kf,kw. [Implementation keywords] 336077

12 (participation adj3 rate*).ti,ab,kf,kw. [Participation rate keywords] 8875

13 engage*.ti,ab,kf,kw. [Engage keywords] 217125

14 or/7-13 [Aspects (Recruitment) set] 1172057

15 "Health Promotion"/ or ("health promotion*" or "wellness program*" or "health campaign*").ti,ab,kf,kw. [Health promotion ] 106362

16 Population health management/ or "population health management".ti,ab,kf,kw. [Population health management] 739

17 Healthy people programs/ or ((program or programs) adj3 (health or healthy or "health related" or healthcare or "health care" or lifestyle* or "life style*" or wellness)).ti,ab,kw,kf. [ Health programs - subject or keyword terms] 58141

18 ("33677529" or "28103935" or "34668873").ui. [Exemplars] 3

19 or/1-2 [Recruitment + PRISM/RE-AIM/reach ] 608

20 6 and (or/15-16) [Recruitment + Health promotion/PopHealthMgmt ] 3673

21 6 and 14 and (or/15-16) [Recruitment + Aspects + Health promotion/PopHealthMgmt ] 1457

22 6 and 17 [Recruitment + Health programs] 2222

23 6 and 14 and 17 [Recruitment + Aspects + Health programs] 990

24 or/19,21,23 [Final set 1a] 2670

25 or/18,24 [Final set 1a finds exemplars] 2670

26 remove duplicates from 24 [Final set 1a with duplicates removed] 2654

Medline (Ovid) Legend

Field codes: / = Medical Subject Heading (MeSH); ti = article title; ab = abstract; kf = keyword heading word; kw = keyword heading (author keywords); ui = unique identifier; pt = publication type; mp = multi-purpose fields; hw = subject heading word; jw = journal word
Proximity operator: adj# 
Truncation:  *

# Embase (embase.com)

Query Results **No.**

#23 #21 NOT ('conference abstract'/it OR 'conference paper'/it) **2,545**

#22 #21 AND ('conference abstract'/it OR 'conference paper'/it) **904**

#21 #18 OR #19 OR #20 **3,449**

#20 #6 AND #14 AND #17 **2,412**

#19 #6 AND #14 AND (#15 OR #16) **1,671**

#18 #1 OR #2 **858**

#17 'health promotion'/de OR (((program OR programs) NEAR/3 (health OR healthy OR 'health related' OR healthcare OR 'health care' OR lifestyle* OR 'life style*' OR wellness)):ti,ab,kw) **167,180**

#16 'population health management'/de OR 'population health management':ti,ab,kw **1,118**

#15 'health promotion'/de OR 'health promotion*':ti,ab,kw OR 'wellness program*':ti,ab,kw OR 'health campaign*':ti,ab,kw **130,496**

#14 #7 OR #8 OR #9 OR #10 OR #11 OR #12 OR #13 **1,737,768**

#13 engage*:ti,ab,kw **274,488**

#12 (participation NEAR/3 rate*):ti,ab,kw **11,446**

#11 implementation:ti,ab,kw **442,497**

#10 representativeness*:ti,ab,kw **5,486**

#9 reach:ti,ab,kw OR outreach:ti,ab,kw **287,369**

#8 enroll*:ti,ab,kw OR enrollment*:ti,ab,kw **796,802**

#7 recruitment:ti,ab,kw AND reach:ti,ab,kw **3,390**

#6 #3 OR #4 OR #5 **535,742**

#5 ((enrollment OR participation OR recruit* OR recruitment) NEAR/6 (method* OR individuals OR patient* OR participant* OR population OR sample OR strategy OR strategies)):ti,ab,kw **295,548**

#4 ((recruit* OR recruitment) NEAR/6 (active* OR passive* OR reach OR representative OR representativeness)):ti,ab,kw **7,887**

#3 'patient selection'/de OR (((recruit* OR selection*) NEAR/3 (active OR criteria OR engagement* OR method* OR outreach OR patient* OR participant* OR passive OR reach OR 'research subject*' OR 'research volunteer*' OR subject OR subjects OR volunteer OR volunteers)):ti,ab,kw) OR 'selection criteria*':ti,ab,kw OR 'selection for treatment*':ti,ab,kw OR 'subject selection*':ti,ab,kw **396,883**

#2 (recruitment NEAR/9 reach):ti,ab,kw **628**

#1 recruitment:ti,ab,kw AND (prism:ti,ab,kw OR 're aim':ti,ab,kw) **238**

Embase Legend

Field codes: ti = article title; ab = abstract; kw = keyword; de = index (descriptor) term; exp = explosion of index term and narrower terms; mj = focused (descriptor) index term; cl = Embase classification; jt = source title; it = publication type
Proximity operator: NEAR/# 
Truncation: *

# CINAHL Complete (Ebscohost)

Interface - EBSCOhost Research Databases
Search Screen - Advanced Search
Database - CINAHL Complete
Search modes - Boolean/Phrase

| **#** | **Query** | **Results** |
| --- | --- | --- |
| S21 | S18 OR S19 OR S20 | 1,826 |
| S20 | S6 AND S14 AND S17 | 983 |
| S19 | S6 AND S14 AND (S15 OR S16) | 830 |
| S18 | S1 OR S2 | 305 |
| S17 | (MM "Health Initiative 2000") OR (MM "Healthy People 2000") OR (MM "Healthy People 2010") OR (MM "Healthy People 2020") OR TI ((program OR programs) N3 (health OR healthy OR "health related" OR healthcare OR "health care" OR lifestyle* OR "life style*" OR wellness)) OR AB ((program OR programs) N3 (health OR healthy OR "health related" OR healthcare OR "health care" OR lifestyle* OR "life style*" OR wellness)) | 45,982 |
| S16 | (MM "Population Health Management") OR TI ("population health management") OR AB ("population health management") | 1,007 |
| S15 | (MM "Health Promotion") OR TI ("health promotion*" OR "wellness program*" OR "health campaign*") OR AB ("health promotion*" OR "wellness program*" OR "health campaign*") | 63,084 |
| S14 | S7 OR S8 OR S9 OR S10 OR S11 OR S12 OR S13 | 424,761 |
| S13 | TI engage* OR AB engage* | 103,218 |
| S12 | TI (participation N3 rate*) OR AB (participation N3 rate*) | 4,105 |
| S11 | TI implementation OR AB implementation | 136,478 |
| S10 | TI representativeness* OR AB representativeness* | 1,458 |
| S9 | TI (reach OR outreach) OR AB (reach OR outreach) | 55,050 |
| S8 | TI (enroll* OR enrollment*) OR AB (enroll* OR enrollment*) | 151,020 |
| S7 | TI (Recruitment AND reach) OR AB (Recruitment AND reach) | 907 |
| S6 | S3 OR S4 OR S5 | 117,962 |
| S5 | TI ( ((enrollment OR participation OR recruit* OR recruitment) N6 (method* OR individuals OR patient* OR participant* OR population OR sample OR strategy OR strategies)) ) OR AB ( ((enrollment OR participation OR recruit* OR recruitment) N6 (method* OR individuals OR patient* OR participant* OR population OR sample OR strategy OR strategies)) ) | 85,388 |
| S4 | TI ( ((Recruit* OR Recruitment) N6 (active* OR passive* OR reach OR representative OR representativeness)) ) OR AB ( ((Recruit* OR Recruitment) N6 (active* OR passive* OR reach OR representative OR representativeness)) ) | 1,993 |
| S3 | (MM "Patient Selection") OR TI (((recruit* OR selection*) N3 (active OR criteria OR engagement* OR method* OR outreach OR patient* OR participant* OR passive OR reach OR "research subject*" OR "research volunteer*" OR subject OR subjects OR volunteer OR volunteers)) OR "selection criteria*" OR "selection for treatment*" OR "subject selection*") OR AB (((recruit* OR selection*) N3 (active OR criteria OR engagement* OR method* OR outreach OR patient* OR participant* OR passive OR reach OR "research subject*" OR "research volunteer*" OR subject OR subjects OR volunteer OR volunteers)) OR "selection criteria*" OR "selection for treatment*" OR "subject selection*") | 74,617 |
| S2 | TI (Recruitment N9 reach) OR AB (Recruitment N9 reach) | 261 |
| S1 | TI (Recruitment AND (PRISM OR RE-AIM)) OR AB (Recruitment AND (PRISM OR RE-AIM)) | 45 |

CINAHL legend
Field codes: TI = title; AB= abstract; MM = CINAHL Exact Subject Heading (Major only);
Proximity operator: N# 
Truncation:  *

# APA PsycINFO (Ebscohost)

Interface - EBSCOhost Research Databases
Search Screen - Advanced Search
Database - APA PsycInfo

Search modes - Boolean/Phrase

| **#** | **Query** | **Results** |
| --- | --- | --- |
| S21 | S18 OR S19 OR S20 | 1,347 |
| S20 | S6 AND S14 AND S17 | 573 |
| S19 | S6 AND S14 AND (S15 OR S16) | 812 |
| S18 | S1 OR S2 | 211 |
| S17 | DE "Public Health Campaigns" OR TI ((program OR programs) N3 (health OR healthy OR "health related" OR healthcare OR "health care" OR lifestyle* OR "life style*" OR wellness)) OR AB ((program OR programs) N3 (health OR healthy OR "health related" OR healthcare OR "health care" OR lifestyle* OR "life style*" OR wellness)) OR KW ((program OR programs) N3 (health OR healthy OR "health related" OR healthcare OR "health care" OR lifestyle* OR "life style*" OR wellness)) | 28,635 |
| S16 | TI ("population health management") OR AB ("population health management") OR KW ("population health management") | 86 |
| S15 | DE "Health Promotion" OR TI ("health promotion*" OR "wellness program*" OR "health campaign*") OR AB ("health promotion*" OR "wellness program*" OR "health campaign*") OR KW ("health promotion*" OR "wellness program*" OR "health campaign*") | 45,454 |
| S14 | S7 OR S8 OR S9 OR S10 OR S11 OR S12 OR S13 | 427,431 |
| S13 | TI engage* OR AB engage* OR KW engage* | 213,949 |
| S12 | TI (participation N3 rate*) OR AB (participation N3 rate*) OR KW (participation N3 rate*) | 3,614 |
| S11 | TI implementation OR AB implementation OR KW implementation | 110,536 |
| S10 | TI representativeness* OR AB representativeness* OR KW representativeness* | 2,240 |
| S9 | TI (reach or outreach) OR AB (reach or outreach) OR KW (reach or outreach) | 55,121 |
| S8 | TI (enroll* OR enrollment*) OR AB (enroll* OR enrollment*) OR KW (enroll* OR enrollment*) | 68,156 |
| S7 | TI (Recruitment AND reach) OR AB (Recruitment AND reach) OR KW (Recruitment AND reach) | 689 |
| S6 | S3 OR S4 OR S5 | 85,745 |
| S5 | TI ( ((enrollment OR participation OR recruit* OR recruitment) N6 (method* OR individuals OR patient* OR participant* OR population OR sample OR strategy OR strategies)) ) OR AB ( ((enrollment OR participation OR recruit* OR recruitment) N6 (method* OR individuals OR patient* OR participant* OR population OR sample OR strategy OR strategies)) ) OR KW ( ((enrollment OR participation OR recruit* OR recruitment) N6 (method* OR individuals OR patient* OR participant* OR population OR sample OR strategy OR strategies)) ) | 65,465 |
| S4 | TI ( ((Recruit* OR Recruitment) N6 (active* OR passive* OR reach OR representative OR representativeness)) ) OR AB ( ((Recruit* OR Recruitment) N6 (active* OR passive* OR reach OR representative OR representativeness)) ) OR KW ( ((Recruit* OR Recruitment) N6 (active* OR passive* OR reach OR representative OR representativeness)) ) | 1,749 |
| S3 | (DE "Patient Selection") OR TI (((recruit* OR selection*) N3 (active OR criteria OR engagement* OR method* OR outreach OR patient* OR participant* OR passive OR reach OR "research subject*" OR "research volunteer*" OR subject OR subjects OR volunteer OR volunteers)) OR "selection criteria*" OR "selection for treatment*" OR "subject selection*") OR AB (((recruit* OR selection*) N3 (active OR criteria OR engagement* OR method* OR outreach OR patient* OR participant* OR passive OR reach OR "research subject*" OR "research volunteer*" OR subject OR subjects OR volunteer OR volunteers)) OR "selection criteria*" OR "selection for treatment*" OR "subject selection*") OR KW (((recruit* OR selection*) N3 (active OR criteria OR engagement* OR method* OR outreach OR patient* OR participant* OR passive OR reach OR "research subject*" OR "research volunteer*" OR subject OR subjects OR volunteer OR volunteers)) OR "selection criteria*" OR "selection for treatment*" OR "subject selection*") | 48,993 |
| S2 | TI (Recruitment N9 reach) OR AB (Recruitment N9 reach) OR KW (Recruitment N9 reach) | 186 |
| S1 | TI (Recruitment AND (PRISM OR RE-AIM)) OR AB (Recruitment AND (PRISM OR RE-AIM)) OR KW (Recruitment AND (PRISM OR RE-AIM)) | 25 |

PsycInfo Legend
Field codes: TI = title; AB = abstract; KW = keyword; DE = Subject Heading or Keyword (Phrase indexed)
Proximity operator: N# 
Truncation:  *

# ProQuest Dissertations and Theses Global (ProQuest)

noft((Recruitment AND (PRISM OR RE-AIM)) OR (Recruitment NEAR/9 reach)) OR (noft((((recruit* OR selection*) NEAR/3 (active OR criteria OR engagement OR method* OR outreach OR patient OR patients OR participant or participants OR passive OR reach OR "research subject" OR "research subjects" OR "research volunteer" OR "research volunteers" OR subject OR subjects OR volunteer OR volunteers)) OR "selection criteria*" OR "selection for treatment" OR "selection for treatments" OR "subject selection") OR ((Recruit* OR Recruitment) NEAR/6 (active* OR passive* OR reach OR representative OR representativeness)) OR ((enrollment OR participation OR recruit* OR recruitment) NEAR/6 (method* OR individuals OR patient OR patients OR participant or participants OR population OR sample OR strategy OR strategies))) AND noft((Recruitment AND reach) OR enroll* OR enrollment* OR reach OR outreach OR representativeness* OR implementation OR (participation NEAR/3 rate*) OR engage*) AND noft("health promotion*" OR "wellness program*" OR "health campaign*" OR "population health management" OR ((program OR programs) NEAR/3 (health OR healthy OR "health related" OR healthcare OR "health care" OR lifestyle* OR "life style*" OR wellness))))

436 results

ProQuest Legend

Field codes: NOFT = Anywhere except full text
Proximity operator: NEAR/# 
Truncation:  *
