# Supplementary material for: Identifying recruitment strategies to improve the reach of evidence-based health promotion, disease prevention, and disease self-management interventions: a scoping review
Source: Front Public Health. 2025 Apr 1;13:1515042. doi: 10.3389/fpubh.2025.1515042 (PMC12023269; doi:10.3389/fpubh.2025.1515042)
Supplement: Supplementary file 3 [file Supplementary_file_3.docx]

Appendix C: Bibliography of Included Studies

*Note: Four articles merged as two studies: Mills 1996 and Mills 2001; Kozica 2015 and Kozica 2015.*

1. Adams R, Hebert CJ, McVey L, Williams R. Implementation of the YMCA Diabetes Prevention Program throughout an Integrated Health System: A Translational Study. *Permanente Journal*. 2016;20(4):15-241. doi: 10.7812/TPP/15-241.

2. Alexander GL, Divine GW, Couper MP, et al. Effect of incentives and mailing features on online health program enrollment. *Am J Prev Med*. 2008;34(5):382-8. doi: 10.1016/j.amepre.2008.01.028.

3. Bajraktari S, Zingmark M, Pettersson B, Rosendahl E, Lundin-Olsson L, Sandlund M. Reaching Older People with a Digital Fall Prevention Intervention in a Swedish Municipality Context-an Observational Study. *Frontiers in Public Health*. 2022;10:857652. doi: 10.3389/fpubh.2022.857652.

4. Bayley A, Stahl D, Ashworth M, et al. Response bias to a randomised controlled trial of a lifestyle intervention in people at high risk of cardiovascular disease: a cross-sectional analysis. *BMC Public Health*. 2018;18(1):1092. doi: 10.1186/s12889-018-5939-y.

5. Bean C, Dineen T, Locke SR, Bouvier B, Jung ME. An Evaluation of the Reach and Effectiveness of a Diabetes Prevention Behaviour Change Program Situated in a Community Site. *Canadian Journal of Diabetes*. 2021;45(4):360-368. doi: 10.1016/j.jcjd.2020.10.006.

6. Befort CA, Kurz D, VanWormer JJ, Ellerbeck EF. Recruitment and reach in a pragmatic behavioral weight loss randomized controlled trial: implications for real-world primary care practice. *BMC Fam Pract*. 2020;21(1):47. doi: 10.1186/s12875-020-01117-w.

7. Benedetti TRB, Rech CR, Konrad LM, Almeida FA, Brito FA, Chodzko-Zajko W, Schwingel A. Re-thinking Physical Activity Programs for Older Brazilians and the Role of Public Health Centers: A Randomized Controlled Trial Using the RE-AIM Model. *Frontiers in Public Health*. 2020;8:48. doi: 10.3389/fpubh.2020.00048.

8. Bracken K, Hague W, Keech A, et al. Recruitment of men to a multi-centre diabetes prevention trial: An evaluation of traditional and online promotional strategies. *Trials*. 2019;20(1)doi: 10.1186/s13063-019-3485-2.

9. Brewer LC, Jenkins S, Lackore K, et al. mHealth Intervention Promoting Cardiovascular Health Among African-Americans: Recruitment and Baseline Characteristics of a Pilot Study. *JMIR Research Protocols*. 2018;7(1):e31. doi: 10.2196/resprot.8842.

10. Brierley ML, Smith LR, Chater AM, Bailey DP. A-REST (Activity to Reduce Excessive Sitting Time): A Feasibility Trial to Reduce Prolonged Sitting in Police Staff. *International Journal of Environmental Research & Public Health*. 2022;19(15):27. doi: 10.3390/ijerph19159186.

11. Brown SD, Lee K, Schoffman DE, King AC, Crawley LM, Kiernan M. Minority recruitment into clinical trials: experimental findings and practical implications. *Contemp Clin Trials*. 2012;33(4):620-3. doi: 10.1016/j.cct.2012.03.003.

12. Brunisholz KD, Kim J, Savitz LA, et al. A Formative Evaluation of a Diabetes Prevention Program Using the RE-AIM Framework in a Learning Health Care System, Utah, 2013-2015. *Prev Chronic Dis*. 2017;14:E58. doi: 10.5888/pcd14.160556.

13. Carter A, Humphreys L, Snowdon N, et al. Participant recruitment into a randomised controlled trial of exercise therapy for people with multiple sclerosis. *Trials*. 2015;16:468. doi: 10.1186/s13063-015-0996-3.

14. Chinn DJ, White M, Howel D, Harland JO, Drinkwater CK. Factors associated with non-participation in a physical activity promotion trial. *Public Health*. 2006;120(4):309-19.

15. Chow EJ, Baldwin LM, Hagen AM, et al. Communicating health information and improving coordination with primary care (CHIIP): Rationale and design of a randomized cardiovascular health promotion trial for adult survivors of childhood cancer. *Contemp Clin Trials*. 2020;89:105915. doi: 10.1016/j.cct.2019.105915.

16. Clark AF, Wilk P, Mitchell CA, Smith C, Archer J, Gilliland JA. Examining How Neighborhood Socioeconomic Status, Geographic Accessibility, and Informational Accessibility Influence the Uptake of a Free Population-Level Physical Activity Intervention for Children. *Am J Health Promot*. 2018;32(2):315-324. doi: 10.1177/0890117117718433.

17. Coughlin JW, Martin LM, Zhao D, et al. Electronic Health Record-Based Recruitment and Retention and Mobile Health App Usage: Multisite Cohort Study. *J Med Internet Res*. 2022;24(6):e34191. doi: 10.2196/34191.

18. Crane MM, LaRose JG, Espeland MA, Wing RR, Tate DF. Recruitment of young adults for weight gain prevention: randomized comparison of direct mail strategies. *Trials*. 2016;17(1):282. doi: 10.1186/s13063-016-1411-4.

19. Daley A, Winter H, Grimmett C, McGuinness M, McManus R, MacArthur C. Feasibility of an exercise intervention for women with postnatal depression: a pilot randomised controlled trial. *Br J Gen Pract*. 2008;58(548):178-83.

20. Dettlaff-Dunowska M, Brzeziński M, Zagierska A, Borkowska A, Zagierski M, Szlagatys-Sidorkiewicz A. Changes in Body Composition and Physical Performance in Children with Excessive Body Weight Participating in an Integrated Weight-Loss Programme. *Nutrients*. 2022;14(17):3647. doi: 10.3390/nu14173647.

21. Eakin EG, Bull SS, Riley K, Reeves MM, Gutierrez S, McLaughlin P. Recruitment and retention of Latinos in a primary care-based physical activity and diet trial: The Resources for Health study. *Health Educ Res*. 2007;22(3):361-71.

22. Effoe VS, Katula JA, Kirk JK, et al. The use of electronic medical records for recruitment in clinical trials: findings from the Lifestyle Intervention for Treatment of Diabetes trial. *Trials*. 2016;17(1):496.

23. Estabrooks PA, Bradshaw M, Dzewaltowski DA, Smith-Ray RL. Determining the impact of Walk Kansas: applying a team-building approach to community physical activity promotion. *Ann Behav Med*. 2008;36(1):1-12. doi: 10.1007/s12160-008-9040-0.

24. Felix HC, Adams B, Fausett JK, Krukowski RA, Prewitt TE, West DS. Calculating reach of evidence-based weight loss and memory improvement interventions among older adults attending Arkansas senior centers, 2008-2011. *Prev Chronic Dis*. 2012;9:E63.

25. Franklin PD, Ploutz-Snyder R, Rosenbaum PF, Carey MP, Smith N, Roizen MF. Worksite e-mail health promotion trial: Early lessons. *Eval Program Plann*. 2006;29(4):405-12.

26. Garip G, Morton K, Bridger R, Yardley L. Evaluating the feasibility of a web-based weight loss programme for naval service personnel with excess body weight. *Pilot & Feasibility Studies*. 2017;3:6. doi: 10.1186/s40814-017-0122-2.

27. Ghai NR, Reynolds KD, Xiang AH, et al. Recruitment results among families contacted for an obesity prevention intervention: The Obesity Prevention Tailored for Health Study. *Trials*. 2014;15:463. doi: 10.1186/1745-6215-15-463.

28. Glasgow RE, Nelson CC, Kearney KA, et al. Reach, engagement, and retention in an internet-based weight loss program in a multi-site randomized controlled trial. *J Med Internet Res*. 2007;9(2):e11. doi: 10.2196/jmir.9.2.e11.

29. Glasgow RE, Toobert DJ. Brief, computer-assisted diabetes dietary self-management counseling: Effects on behavior, physiologic outcomes, and quality of life. *Med Care*. 2000;38(11):1062-1073. doi: 10.1097/00005650-200011000-00002.

30. Gopalan A, Paramanund J, Shaw PA, et al. Randomised controlled trial of alternative messages to increase enrolment in a healthy food programme among individuals with diabetes. *BMJ Open*. 2016;6(11):e012009. doi: 10.1136/bmjopen-2016-012009.

31. Guertler D, Meyer C, Dorr M, et al. Reach of Individuals at Risk for Cardiovascular Disease by Proactive Recruitment Strategies in General Practices, Job Centers, and Health Insurance. *Int J Behav Med*. 2017;24(1):153-160. doi: 10.1007/s12529-016-9584-5.

32. Harden SM, Fanning JT, Motl RW, McAuley E, Estabrooks PA. Determining the reach of a home-based physical activity program for older adults within the context of a randomized controlled trial. *Health Educ Res*. 2014;29(5):861-9. doi: 10.1093/her/cyu049.

33. Hirsch SH, Mayer-Oakes A, Schweitzer S, Atchison KA, Lubben JE, DeJong F. Enrolling community physicians and their patients in a study of prevention in the elderly. *Public Health Rep*. 1992;107(2):142-9.

34. Horowitz CR, Brenner BL, Lachapelle S, Amara DA, Arniella G. Effective recruitment of minority populations through community-led strategies. *Am J Prev Med*. 2009;37(6 Suppl 1):S195-200. doi: 10.1016/j.amepre.2009.08.006.

35. Jago R, Tibbitts B, Porter A, et al. A revised teaching assistant-led extracurricular physical activity programme for 8- to 10-year-olds: The Action 3:30R feasibility cluster RCT. *Public Health Research*. 2019;12(19):12. doi: 10.3310/phr07190.

36. Jalkanen K, Jarvenpaa R, Tilles-Tirkkonen T, et al. Comparison of Communication Channels for Large-Scale Type 2 Diabetes Risk Screening and Intervention Recruitment: Empirical Study. *JMIR Diabetes*. 2021;6(3):e21356. doi: 10.2196/21356.

37. Johnson EM, Oddone EZ, Van Treese K, et al. Implementing evidence-based telephone coaching for health behavior program enrollment: A quality improvement project. *Families, Systems, & Health*. 2022;17:17. doi: 10.1037/fsh0000758.

38. Jong ST, Croxson CHD, Foubister C, et al. Reach, Recruitment, Dose, and Intervention Fidelity of the GoActive School-Based Physical Activity Intervention in the UK: A Mixed-Methods Process Evaluation. *Children*. 2020;7(11):17. doi: 10.3390/children7110231.

39. Kerry SM, Morgan KE, Limb E, et al. Interpreting population reach of a large, successful physical activity trial delivered through primary care. *BMC Public Health*. 2018;18(1):170. doi: 10.1186/s12889-018-5034-4.

40. Kirley K, Khan T, Aquino G, Brown A, Meier S, Chambers N, O'Connell C. Using a certified electronic health record technology platform to screen, test and refer patients with prediabetes. *JAMIA Open*. 2021;4(4):ooab101. doi: 10.1093/jamiaopen/ooab101.

41. Kozica SL, Harrison CL, Teede HJ, Ng S, Moran LJ, Lombard CB. Engaging rural women in healthy lifestyle programs: insights from a randomized controlled trial. *Trials*. 2015;16:413. doi: 10.1186/s13063-015-0860-5.

42. Kozica SL, Lombard CB, Ilic D, Ng S, Harrison CL, Teede HJ. Acceptability of delivery modes for lifestyle advice in a large scale randomised controlled obesity prevention trial. *BMC Public Health*. 2015;15:699. doi: 10.1186/s12889-015-1995-8.

43. Lawlor ER, Cupples ME, Donnelly M, Tully MA. Promoting physical activity among community groups of older women in socio-economically disadvantaged areas: randomised feasibility study. *Trials*. 2019;20(1):234. doi: 10.1186/s13063-019-3312-9.

44. Lewis ZH, Ottenbacher KJ, Fisher SR, et al. The feasibility and RE-AIM evaluation of the TAME health pilot study. *International Journal of Behavioral Nutrition & Physical Activity*. 2017;14(1):106. doi: 10.1186/s12966-017-0560-5.

45. Linnan L, Tate DF, Harrington CB, et al. Organizational- and employee-level recruitment into a worksite-based weight loss study. *Clinical Trials*. 2012;9(2):215-25. doi: 10.1177/1740774511432554.

46. Linnan LA, Emmons KM, Klar N, Fava JL, LaForge RG, Abrams DB. Challenges to improving the impact of worksite cancer prevention programs: comparing reach, enrollment, and attrition using active versus passive recruitment strategies. *Ann Behav Med*. 2002;24(2):157-66.

47. Liu J, Wilcox S, Wingard E, Burgis J, Schneider L, Dahl A. Strategies and Challenges in Recruiting Pregnant Women with Elevated Body Mass Index for a Behavioral Lifestyle Intervention. *Women's Health Reports*. 2020;1(1):556-565. doi: 10.1089/whr.2020.0089.

48. Liu S, Hodgson C, Zbib AM, Payne AY, Nolan RP. The effectiveness of loyalty rewards to promote the use of an Internet-based heart health program. *J Med Internet Res*. 2014;16(7):e163. doi: 10.2196/jmir.3458.

49. Long DA, Sheehan P. A case study of population health improvement at a Midwest regional hospital employer. *Population Health Management*. 2010;13(3):163-73. doi: 10.1089/pop.2008.0034.

50. Madsen K, Garber A, Martin M, Gonzaga M, Linchey J. The feasibility of a physical activity referral network for pediatric obesity. *Childhood Obesity*. 2014;10(2):169-174. doi: 10.1089/chi.2013.0118.

51. Markert J, Alff F, Zschaler S, Gausche R, Kiess W, Bluher S. Prevention of childhood obesity: recruiting strategies via local paediatricians and study protocol for a telephone-based counselling programme. *Obes Res Clin Pract*. 2013;7(6):e476-86. doi: 10.1016/j.orcp.2012.07.008.

52. Mas-Alos S, Planas-Anzano A, Peirau-Teres X, Real-Gatius J, Galindo-Ortego G. Feasibility Assessment of the Let's Walk Programme (CAMINEM): Exercise Training and Health Promotion in Primary Health-Care Settings. *International Journal of Environmental Research & Public Health*. 2021;18(6):19. doi: 10.3390/ijerph18063192.

53. McEachan RRC, Santorelli G, Bryant M, et al. The HAPPY (Healthy and Active Parenting Programmme for early Years) feasibility randomised control trial: acceptability and feasibility of an intervention to reduce infant obesity. *BMC Public Health*. 2016;16(1):1-15. doi: 10.1186/s12889-016-2861-z.

54. Mills KM, Stewart AL, King AC, Roitz K, Sepsis PG, Ritter PL, Bortz WM, 2nd. Factors associated with enrollment of older adults into a physical activity promotion program. *J Aging Health*. 1996;8(1):96-113.

55. Mills KM, Stewart AL, McLellan BY, Verboncoeur CJ, King AC, Brown BW. Evaluation of enrollment bias in a physical-activity-promotion program for seniors. *J Aging Phys Act*. 2001;9(4):398-413. doi: 10.1123/japa.9.4.398.

56. Mullane SL, Rydell SA, Larouche ML, et al. Enrollment Strategies, Barriers to Participation, and Reach of a Workplace Intervention Targeting Sedentary Behavior. *Am J Health Promot*. 2019;33(2):225-236. doi: 10.1177/0890117118784228.

57. Oddone EZ, Gierisch JM, Sanders LL, et al. A Coaching by Telephone Intervention on Engaging Patients to Address Modifiable Cardiovascular Risk Factors: A Randomized Controlled Trial. *J Gen Intern Med*. 2018;33(9):1487-1494. doi: 10.1007/s11606-018-4398-6.

58. Okhomina VI, Seals SR, Marshall GD, Jr. Recruitment and enrollment of African Americans into health promoting programs: the effects of health promoting programs on cardiovascular disease risk study. *Ethn Health*. 2020;25(6):825-834. doi: 10.1080/13557858.2018.1458074.

59. Olij BF, Erasmus V, Barmentloo LM, et al. Evaluation of Implementing a Home-Based Fall Prevention Program among Community-Dwelling Older Adults. *International Journal of Environmental Research & Public Health*. 2019;16(6):26. doi: 10.3390/ijerph16061079.

60. Parkinson MD, Hammonds T, Keyser DJ, Wheeler JR, Peele PB. Impact of Physician Referral to Health Coaching on Patient Engagement and Health Risks: An Observational Study of UPMC's Prescription for Wellness. *Am J Health Promot*. 2020;34(4):366-375. doi: 10.1177/0890117119900588.

61. Parra-Medina D, D'Antonio A, Smith SM, Levin S, Kirkner G, Mayer-Davis E, Power s. Successful recruitment and retention strategies for a randomized weight management trial for people with diabetes living in rural, medically underserved counties of South Carolina: the POWER study. *J Am Diet Assoc*. 2004;104(1):70-5.

62. Partridge SR, Balestracci K, Wong AT, et al. Effective Strategies to Recruit Young Adults into the TXT2BFiT mHealth Randomized Controlled Trial for Weight Gain Prevention. *JMIR Research Protocols*. 2015;4(2):e66. doi: 10.2196/resprot.4268.

63. Peck LE, Sharpe PA, Burroughs EL, Granner ML. Recruitment strategies and costs for a community-based physical activity program. *Health Promotion Practice*. 2008;9(2):191-8.

64. Peels DA, Bolman C, Golsteijn RH, De Vries H, Mudde AN, van Stralen MM, Lechner L. Differences in reach and attrition between Web-based and print-delivered tailored interventions among adults over 50 years of age: clustered randomized trial. *J Med Internet Res*. 2012;14(6):e179. doi: 10.2196/jmir.2229.

65. Porter G, Michaud TL, Schwab RJ, Hill JL, Estabrooks PA. Reach Outcomes and Costs of Different Physician Referral Strategies for a Weight Management Program Among Rural Primary Care Patients: Type 3 Hybrid Effectiveness-Implementation Trial. *JMIR Formative Research*. 2021;5(10):e28622. doi: 10.2196/28622.

66. Ramsay JE, Hogan CK, Janevic MR, Courser RR, Allgood KL, Connell CM. Comparison of recruitment strategies for engaging older minority adults: Results from Take Heart. *The Journals of Gerontology: Series A: Biological Sciences and Medical Sciences*. 2020;75(5):922-928. doi: 10.1093/gerona/glz112.

67. Robroek SJ, Lindeboom DE, Burdorf A. Initial and sustained participation in an internet-delivered long-term worksite health promotion program on physical activity and nutrition. *J Med Internet Res*. 2012;14(2):e43. doi: 10.2196/jmir.1788.

68. Samuel-Hodge CD, Garcia BA, Johnston LF, et al. Rationale, design, and sample characteristics of a practical randomized trial to assess a weight loss intervention for low-income women: The Weight-Wise II Program. *Contemp Clin Trials*. 2012;33(1):93-103. doi: 10.1016/j.cct.2011.08.009.

69. Sanchez A, Silvestre C, Campo N, Grandes G, Pre DErg. Type-2 diabetes primary prevention program implemented in routine primary care: a process evaluation study. *Trials*. 2016;17(1):254. doi: 10.1186/s13063-016-1379-0.

70. Santoyo-Olsson J, Cabrera J, Freyre R, et al. An innovative multiphased strategy to recruit underserved adults into a randomized trial of a community-based diabetes risk reduction program. *Gerontologist*. 2011;51 Suppl 1:S82-93. doi: 10.1093/geront/gnr026.

71. Sharpe PA, Stucker J, Wilcox S, Liese AD, Bell BA. Recruitment and retention for the evaluation of a healthy food initiative in economically disadvantaged, majority African American communities. *Family & Community Health: The Journal of Health Promotion & Maintenance*. 2021;44(1):43-51. doi: 10.1097/FCH.0000000000000259.

72. Snyder DC, Morey MC, Sloane R, et al. Reach out to ENhancE Wellness in Older Cancer Survivors (RENEW): design, methods and recruitment challenges of a home-based exercise and diet intervention to improve physical function among long-term survivors of breast, prostate, and colorectal cancer. *Psychooncology*. 2009;18(4):429-39. doi: 10.1002/pon.1491.

73. Speck RM, Hill RK, Pronk NP, Becker MP, Schmitz KH. Assessment and outcomes of HealthPartners 10,000 Steps program in an academic work site. *Health Promotion Practice*. 2010;11(5):741-50. doi: 10.1177/1524839908330745.

74. Spittaels H, De Bourdeaudhuij I. Who participates in a computer-tailored physical activity program delivered through the Internet? A comparison of participants' and non-participants' characteristics. *International Journal of Behavioral Nutrition & Physical Activity*. 2007;4:39.

75. Stevens M, de Jong J, Lemmink KA. The Groningen Active Living Model, an example of successful recruitment of sedentary and underactive older adults. *Prev Med*. 2008;47(4):398-401. doi: 10.1016/j.ypmed.2008.07.004.

76. Stineman MG, Strumpf N, Kurichi JE, Charles J, Grisso JA, Jayadevappa R. Attempts to reach the oldest and frailest: recruitment, adherence, and retention of urban elderly persons to a falls reduction exercise program. *Gerontologist*. 2011;51 Suppl 1:S59-72. doi: 10.1093/geront/gnr012.

77. Stopponi MA, Alexander GL, McClure JB, et al. Recruitment to a randomized web-based nutritional intervention trial: characteristics of participants compared to non-participants. *J Med Internet Res*. 2009;11(3):e38. doi: 10.2196/jmir.1086.

78. Taradash J, Kramer M, Molenaar D, Arena V, Vanderwood K, Kriska AM. Recruitment for a Diabetes Prevention Program translation effort in a worksite setting. *Contemp Clin Trials*. 2015;41:204-10. doi: 10.1016/j.cct.2015.01.010.

79. Tercyak KP, Donze JR, Prahlad S, Mosher RB, Shad AT. Identifying, recruiting, and enrolling adolescent survivors of childhood cancer into a randomized controlled trial of health promotion: preliminary experiences in the Survivor Health and Resilience Education (SHARE) Program. *J Pediatr Psychol*. 2006;31(3):252-61.

80. Terry PE, Fowles JB, Harvey L. Employee engagement factors that affect enrollment compared with retention in two coaching programs--the ACTIVATE study. *Population Health Management*. 2010;13(3):115-22. doi: 10.1089/pop.2009.0040.

81. Thilsing T, Larsen LB, Sonderlund AL, et al. Effects of a Co-Design-Based Invitation Strategy on Participation in a Preventive Health Check Program: Randomized Controlled Trial. *JMIR Public Health and Surveillance*. 2021;7(3):e25617. doi: 10.2196/25617.

82. Tidwell L, Holland SK, Greenberg J, Malone J, Mullan J, Newcomer R. Community-based nurse health coaching and its effect on fitness participation. *Lippincott's Case Management*. 2004;9(6):267-79.

83. Toobert DJ, Strycker LA, Glasgow RE, Bagdade JD. If you build it, will they come?. Reach and Adoption associated with a comprehensive lifestyle management program for women with type 2 diabetes. *Patient Educ Couns*. 2002;48(2):99-105.

84. Turner CD, Lindsay R, Heisler M. Peer Coaching to Improve Diabetes Self-Management Among Low-Income Black Veteran Men: A Mixed Methods Assessment of Enrollment and Engagement. *Ann Fam Med*. 2021;19(6):532-539. doi: 10.1370/afm.2742.

85. van der Giesen FJ, van Lankveld W, Hopman-Rock M, et al. Exploring the public health impact of an intensive exercise program for patients with rheumatoid arthritis: a dissemination and implementation study. *Arthritis Care Res (Hoboken)*. 2010;62(6):865-72. doi: 10.1002/acr.20138.

86. van Dongen EJ, Duijzer G, Jansen SC, et al. Process evaluation of a randomised controlled trial of a diabetes prevention intervention in Dutch primary health care: the SLIMMER study. *Public Health Nutr*. 2016;19(16):3027-3038.

87. van Holland BJ, Brouwer S, de Boer MR, Reneman MF, Soer R. Process Evaluation of a Workers' Health Surveillance Program for Meat Processing Workers. *Journal of Occupational Rehabilitation*. 2017;27(3):307-318. doi: 10.1007/s10926-016-9657-y.

88. Verburgh M, Verdonk P, Appelman Y, Brood-van Zanten M, Hulshof C, Nieuwenhuijsen K. Workplace Health Promotion Among Ethnically Diverse Women in Midlife with a Low Socioeconomic Position. *Health Educ Behav*. 2022:10901981211071030. doi: 10.1177/10901981211071030.

89. Vermunt PWA, Milder IEJ, Wielaard F, van Oers JAM, Westert GP. An active strategy to identify individuals eligible for type 2 diabetes prevention by lifestyle intervention in Dutch primary care: The APHRODITE study. *Fam Pract*. 2010;27(3):312-319. doi: 10.1093/fampra/cmp100.

90. Vincent D, McEwen MM, Hepworth JT, Stump CS. Challenges and success of recruiting and retention for a culturally tailored diabetes prevention program for adults of Mexican descent. *The Diabetes educator*. 2013;39(2):222-230. doi: 10.1177/0145721713475842.

91. Wages JG, Jackson SF, Bradshaw MH, Chang M, Estabrooks PA. Different strategies contribute to community physical activity program participation in rural versus metropolitan settings. *Am J Health Promot*. 2010;25(1):36-9. doi: 10.4278/ajhp.080729-ARB-143.

92. Ward DS, Vaughn AE, Burney RV, Ostbye T. Recruitment of Family Child Care Homes for an Obesity Prevention Intervention Study. *Contemporary Clinical Trials Communications*. 2016;3:131-138.

93. Ware LJ, Hurling R, Bataveljic O, et al. Rates and determinants of uptake and use of an internet physical activity and weight management program in office and manufacturing work sites in England: cohort study. *J Med Internet Res*. 2008;10(4):e56. doi: 10.2196/jmir.1108.

94. Weston KL, Innerd A, Azevedo LB, Bock S, Batterham AM. Process Evaluation of Project FFAB (Fun Fast Activity Blasts): A Multi-Activity School-Based High-Intensity Interval Training Intervention. *Frontiers in Sports & Active Living*. 2021;3:737900. doi: 10.3389/fspor.2021.737900.

95. Wilson KE, Michaud TL, Almeida FA, et al. Using a population health management approach to enroll participants in a diabetes prevention trial: reach outcomes from the PREDICTS randomized clinical trial. *Transl Behav Med*. 2021;11(5):1066-1077. doi: 10.1093/tbm/ibab010.

96. Withall J, Jago R, Fox KR. The effect a of community-based social marketing campaign on recruitment and retention of low-income groups into physical activity programmes - a controlled before-and-after study. *BMC Public Health*. 2012;12:836. doi: 10.1186/1471-2458-12-836.

97. Xiao H, Adams SR, Goler N, et al. Wellness Coaching for People with Prediabetes: A Randomized Encouragement Trial to Evaluate Outreach Methods at Kaiser Permanente, Northern California, 2013. *Prev Chronic Dis*. 2015;12:E207. doi: 10.5888/pcd12.150251.

98. Yancey AK, Miles OL, McCarthy WJ, Sandoval G, Hill J, Leslie JJ, Harrison GG. Differential response to targeted recruitment strategies to fitness promotion research by African-American women of varying body mass index. *Ethn Dis*. 2001;11(1):115-23.s

99. Yank V, Stafford RS, Rosas LG, Ma J. Baseline reach and adoption characteristics in a randomized controlled trial of two weight loss interventions translated into primary care: a structured report of real-world applicability. *Contemp Clin Trials*. 2013;34(1):126-35. doi: 10.1016/j.cct.2012.10.007.

100. Yeary KHK, Moore PC, Gauss CH, et al. Reach and Adoption of a Randomized Weight Loss Maintenance Trial in Rural African Americans of Faith: The WORD (Wholeness, Oneness, Righteousness, Deliverance). *Am J Health Promot*. 2019;33(4):549-557. doi: 10.1177/0890117118805065.
