# Supplementary material for: Identifying recruitment strategies to improve the reach of evidence-based health promotion, disease prevention, and disease self-management interventions: a scoping review
Source: Front Public Health. 2025 Apr 1;13:1515042. doi: 10.3389/fpubh.2025.1515042 (PMC12023269; doi:10.3389/fpubh.2025.1515042)
Supplement: Supplementary file 4 [file Supplementary_file_4.docx]

Appendix D: Bibliography of Excluded Studies with Reasons

1. Ablah E, Umansky E, Wilcox EA, Usher J, Church J, Barnes V. Innovative recruitment strategies for a comprehensive worksite wellness initiative. *Health Educ Res*. 2019;34(6):569-577. doi: 10.1093/her/cyz030.

Exclusion reason: Recruitment strategy not specified.

2. Aguiar EJ, Morgan PJ, Collins CE, Plotnikoff RC, Young MD, Callister R. Process Evaluation of the Type 2 Diabetes Mellitus PULSE Program Randomized Controlled Trial: Recruitment, Engagement, and Overall Satisfaction. *American Journal of Mens Health*. 2017;11(4):1055-1068. doi: 10.1177/1557988317701783.

Exclusion reason: No denominator or participation rate.

3. Androutsos O, Apostolidou E, Iotova V, et al. Process evaluation design and tools used in a kindergarten-based, family-involved intervention to prevent obesity in early childhood. The ToyBox-study. *Obes Rev*. 2014;15 Suppl 3:74-80. doi: 10.1111/obr.12185.

Exclusion reason: No denominator or participation rate.

4. Atkins CJ, Patterson TL, Roppe BE, Kaplan RM, Sallis JF, Nadar PR. Recruitment issues, health habits, and the decision to participate in a health promotion program. *Am J Prev Med*. 1987;3(2):87-94.

Exclusion reason: No denominator or participation rate.

5. Balis LE, Harden SM. "Replanning" a Statewide Walking Program Through the Iterative Use of the Reach, Effectiveness, Adoption, Implementation, and Maintenance Framework. *Journal of Physical Activity & Health*. 2021;18(10):1310-1317. doi: 10.1123/jpah.2021-0034.

Exclusion reason: Recruitment strategy not specified.

6. Ball K, Abbott G, Wilson M, Chisholm M, Sahlqvist S. How to get a nation walking: reach, retention, participant characteristics and program implications of Heart Foundation Walking, a nationwide Australian community-based walking program. *International Journal of Behavioral Nutrition & Physical Activity*. 2017;14(1):161. doi: 10.1186/s12966-017-0617-5.

Exclusion reason: No denominator or participation rate.

7. Barlow SE, Lorenzi A, Reid A, Huang R, Yudkin JS, Messiah SE. The Implementation and Five-Year Evolution of a Childhood Healthy Weight Program: Making a Health Care-Community Partnership Work. *Childhood Obesity*. 2021;17(7):432-441. doi: 10.1089/chi.2021.0010.

Exclusion reason: No denominator or participation rate.

8. Barratt A, Reznik R, Irwig L, et al. Work-site cholesterol screening and dietary intervention: The Staff Healthy Heart Project. Steering Committee. *Am J Public Health*. 1994;84(5):779-82.

Exclusion reason: No denominator or participation rate.

9. Bird EL, Biddle MSY, Powell JE. General practice referral of 'at risk' populations to community leisure services: applying the RE-AIM framework to evaluate the impact of a community-based physical activity programme for inactive adults with long-term conditions. *BMC Public Health*. 2019;19(1):1308. doi: 10.1186/s12889-019-7701-5.

Exclusion reason: No denominator or participation rate.

10. Blackwell CS, Foster KA, Isom S, Katula JA, Vitolins MZ, Rosenberger EL, Goff DC, Jr. Healthy Living Partnerships to Prevent Diabetes: recruitment and baseline characteristics. *Contemp Clin Trials*. 2011;32(1):40-9. doi: 10.1016/j.cct.2010.10.006.

Exclusion reason: No denominator or participation rate.

11. Branscum P, Sharma M, Wang LL, Wilson B, Rojas-Guyler L. A process evaluation of a social cognitive theory-based childhood obesity prevention intervention: The Comics for Health program. *Health Promotion Practice*. 2013;14(2):189-98. doi: 10.1177/1524839912437790.

Exclusion reason: No denominator or participation rate.

12. Brantley PJ, Guan W, Brock R, Zhang D, Hu G. HEADS UP: Design and Methods of a Louisiana State-Funded Surgical and Non-Surgical Weight Loss Program. *Int J Environ Res Public Health*. 2020;17(9):26. doi: 10.3390/ijerph17092999.

Exclusion reason: Recruitment strategy not specified.

13. Buis LR, Hirzel L, Turske SA, Des Jardins TR, Yarandi H, Bondurant P. Use of a text message program to raise type 2 diabetes risk awareness and promote health behavior change (part I): assessment of participant reach and adoption. *J Med Internet Res*. 2013;15(12):e281. doi: 10.2196/jmir.2928.

Exclusion reason: No denominator or participation rate.

14. Byrd-Bredbenner C, Delaney C, Martin-Biggers J, Koenings M, Quick V. The marketing plan and outcome indicators for recruiting and retaining parents in the HomeStyles randomized controlled trial. *Trials*. 2017;18(1)doi: 10.1186/s13063-017-2262-3.

Exclusion reason: No denominator or participation rate.

15. Corder K, Sharp SJ, Jong ST, et al. Effectiveness and cost-effectiveness of the GoActive intervention to increase physical activity among UK adolescents: A cluster randomised controlled trial. *PLoS Med*. 2020;17(7):e1003210. doi: 10.1371/journal.pmed.1003210.

Exclusion reason: Recruitment strategy not specified.

16. Corr M, McMullen J, Morgan PJ, Barnes A, Murtagh EM. Supporting Our Lifelong Engagement: Mothers and Teens Exercising (SOLE MATES); a feasibility trial. *Women Health*. 2020;60(6):618-635. doi: 10.1080/03630242.2019.1688446.

Exclusion reason: No denominator or participation rate.

17. Devine CM, Maley M, Farrell TJ, Warren B, Sadigov S, Carroll J. Process evaluation of an environmental walking and healthy eating pilot in small rural worksites. *Eval Program Plann*. 2012;35(1):88-96. doi: 10.1016/j.evalprogplan.2011.08.002.

Exclusion reason: Recruitment strategy not specified.

18. Dlugonski D, Schwab L, DuBose KD. Feasibility of the Mothers and Daughters Moving Together Physical Activity Intervention. *Pediatr Exerc Sci*. 2022;34(2):84-92. doi: 10.1123/pes.2021-0024.

Exclusion reason: No denominator or participation rate.

19. Dokkum NF, Koekenbier RH, van den Broek IV, et al. Keeping participants on board: increasing uptake by automated respondent reminders in an Internet-based chlamydia screening in the Netherlands. *BMC Public Health*. 2012;12:176. doi: 10.1186/1471-2458-12-176.

Exclusion reason: Intervention not in content area.

20. Estabrooks P, You W, Hedrick V, Reinholt M, Dohm E, Zoellner J. A pragmatic examination of active and passive recruitment methods to improve the reach of community lifestyle programs: The Talking Health Trial. *International Journal of Behavioral Nutrition & Physical Activity*. 2017;14(1):7. doi: 10.1186/s12966-017-0462-6.

Exclusion reason: No denominator or participation rate.

21. Fazzino TL, Rose GL, Pollack SM, Helzer JE. Recruiting U.S. and Canadian college students via social media for participation in a web-based brief intervention study. *Journal of Studies on Alcohol & Drugs*. 2015;76(1):127-32.

Exclusion reason: No denominator or participation rate.

22. Fink J. *Assessing the impact of an incentivized employee wellness program on participation and weight*. The University of Wisconsin - Milwaukee; 2015.

Exclusion reason: Thesis/Dissertation.

23. Frampton SB, Brochu S, Murray J. Creative strategies for promotion of worksite health services: The Employee Ambassador Program. *Am J Health Promot*. 1996;11(1):10-11. doi: 10.4278/0890-1171-11.1.10.

Exclusion reason: Intervention not in content area.

24. Franz C, Atwood S, Orav EJ, et al. Community-based outreach associated with increased health utilization among Navajo individuals living with diabetes: a matched cohort study. *BMC Health Serv Res*. 2020;20(1):460. doi: 10.1186/s12913-020-05231-4.

Exclusion reason: Intervention not in content area.

25. Gary TL, Hill-Briggs F, Batts-Turner M, Brancati FL. Translational research principles of an effectiveness trial for diabetes care in an urban African American population. *Diabetes Educ*. 2005;31(6):880-9.

Exclusion reason: No denominator or participation rate.

26. Gillespie J, Hughes AR, Gibson AM, Haines J, Taveras EM, Stewart L, Reilly JJ. Healthy Habits, Happy Homes Scotland (4HS) feasibility study: Translation of a home-based early childhood obesity prevention intervention evaluated using RE-AIM framework. *Public Health in Practice*. 2020;1:100026. doi: 10.1016/j.puhip.2020.100026.

Exclusion reason: No denominator or participation rate.

27. Gladback C, Oprinovich S. Prediabetes screening intervention used to promote a lifestyle change program. *Journal of the American Pharmacists Association: JAPhA*. 2021;61(4S):S135-S139. doi: 10.1016/j.japh.2021.01.013.

Exclusion reason: No denominator or participation rate.

28. Graham LF, Scott L, Lopeyok E, Douglas H, Gubrium A, Buchanan D. Outreach Strategies to Recruit Low-Income African American Men to Participate in Health Promotion Programs and Research: Lessons from the Men of Color Health Awareness (MOCHA) Project. *American Journal of Mens Health*. 2018;12(5):1307-1316. doi: 10.1177/1557988318768602.

Exclusion reason: No denominator or participation rate.

29. Griffin TL, Clarke JL, Lancashire ER, et al. Waves study trial i. Process evaluation results of a cluster randomised controlled childhood obesity prevention trial: the WAVES study. *BMC Public Health*. 2017;17(1):681. doi: 10.1186/s12889-017-4690-0.

Exclusion reason: No denominator or participation rate.

30. Halbert CH, Kumanyika S, Bowman M, et al. Participation rates and representativeness of African Americans recruited to a health promotion program. *Health Educ Res*. 2010;25(1):6-13. doi: 10.1093/her/cyp057.

Exclusion reason: No denominator or participation rate.

31. Hammersley ML, Wyse RJ, Jones RA, et al. Telephone and Web-Based Delivery of Healthy Eating and Active Living Interventions for Parents of Children Aged 2 to 6 Years: Mixed Methods Process Evaluation of the Time for Healthy Habits Translation Trial. *J Med Internet Res*. 2022;24(5):e35771. doi: 10.2196/35771.

Exclusion reason: No denominator or participation rate.

32. Hanson S, Cross J, Jones A. Promoting physical activity interventions in communities with poor health and socio-economic profiles: A process evaluation of the implementation of a new walking group scheme. *Soc Sci Med*. 2016;169:77-85. doi: 10.1016/j.socscimed.2016.09.035.

Exclusion reason: No denominator or participation rate.

33. Hare ME, Coday M, Williams NA, et al. Methods and baseline characteristics of a randomized trial treating early childhood obesity: The Positive Lifestyles for Active Youngsters (Team PLAY) trial. *Contemp Clin Trials*. 2012;33(3):534-49. doi: 10.1016/j.cct.2012.02.003.

Exclusion reason: No denominator or participation rate.

34. Harris L, Hankey C, Jones N, et al. Process evaluation of a cluster-randomised controlled trial of multi-component weight management programme in adults with intellectual disabilities and obesity. *J Intellect Disabil Res*. 2019;63(1):49-63. doi: 10.1111/jir.12563.

Exclusion reason: No denominator or participation rate.

35. Hartman MA, Nierkens V, Cremer SW, Stronks K, Verhoeff AP. A process evaluation: does recruitment for an exercise program through ethnically specific channels and key figures contribute to its reach and receptivity in ethnic minority mothers? *BMC Public Health*. 2013;13:768. doi: 10.1186/1471-2458-13-768.

Exclusion reason: No denominator or participation rate.

36. Haynes A, Sherrington C, Wallbank G, et al. Using self-determination theory to understand and improve recruitment for the Coaching for Healthy Ageing (CHAnGE) trial. *PLoS One*. 2021;16(11):e0259873. doi: 10.1371/journal.pone.0259873.

Exclusion reason: No denominator or participation rate.

37. Healy GN, Winkler EAH, Goode AD. A RE-AIM evaluation in early adopters to iteratively improve the online BeUpstanding TM program supporting workers to sit less and move more. *BMC Public Health*. 2021;21(1):1916. doi: 10.1186/s12889-021-11993-1.

Exclusion reason: No denominator or participation rate.

38. Hildebrand M, Neufeld P. Recruiting older adults into a physical activity promotion program: Active Living Every Day offered in a naturally occurring retirement community. *Gerontologist*. 2009;49(5):702-10. doi: 10.1093/geront/gnp072.

Exclusion reason: No denominator or participation rate.

39. Huang H, Mattke S, Batorsky B, Miles J, Liu H, Taylor E. Incentives, Program Configuration, and Employee Uptake of Workplace Wellness Programs. *J Occup Environ Med*. 2016;58(1):30-4. doi: 10.1097/JOM.0000000000000613.

Exclusion reason: Not experimental or quasi-experimental.

40. Hutto B, Saunders RP, Wilcox S, et al. Pathways of influences leading to adoption of the Faith, Activity and Nutrition (FAN) program in a statewide initiative. *Eval Program Plann*. 2021;87:101941. doi: 10.1016/j.evalprogplan.2021.101941.

Exclusion reason: No denominator or participation rate.

41. Kakumanu S, Manns BJ, Tran S, et al. Cost analysis and efficacy of recruitment strategies used in a large pragmatic community-based clinical trial targeting low-income seniors: a comparative descriptive analysis. *Trials*. 2019;20(1):577. doi: 10.1186/s13063-019-3652-5.

Exclusion reason: No denominator or participation rate.

42. Kasson E, Vazquez MM, Doroshenko C, Fitzsimmons-Craft EE, Wilfley DE, Taylor CB, Cavazos-Rehg PA. Exploring Social Media Recruitment Strategies and Preliminary Acceptability of an mHealth Tool for Teens with Eating Disorders. *Int J Environ Res Public Health*. 2021;18(15):28. doi: 10.3390/ijerph18157979.

Exclusion reason: Intervention not in content area.

43. Keller C, Vega-Lopez S, Ainsworth B, Nagle-Williams A, Records K, Permana P, Coonrod D. Social marketing: approach to cultural and contextual relevance in a community-based physical activity intervention. *Health Promot Int*. 2014;29(1):130-40. doi: 10.1093/heapro/das053.

Exclusion reason: No denominator or participation rate.

44. Kennedy BM, Newton RL, Jr., York-Crowe E, Walden HM, Ryan DH, White MA, Williamson DA. Recruiting African American girls and parents for a secondary weight gain prevention study. *J Cult Divers*. 2008;15(4):181-6.

Exclusion reason: No denominator or participation rate.

45. Kluding PM, Singh R, Goetz J, Rucker J, Bracciano S, Curry N. Feasibility and effectiveness of a pilot health promotion program for adults with type 2 diabetes: lessons learned. *Diabetes Educ*. 2010;36(4):595-602. doi: 10.1177/0145721710370718.

Exclusion reason: No denominator or participation rate.

46. Kocourek J. *Effect of a family heart health program on stage of change constructs of exercise and leisure time exercise in family members of patients with heart disease*. University of Ottawa (Canada); 2009.

Exclusion reason: Thesis/Dissertation.

47. Korde LA, Micheli A, Smith AW, et al. Recruitment to a physical activity intervention study in women at increased risk of breast cancer. *BMC Med Res Methodol*. 2009;9:27. doi: 10.1186/1471-2288-9-27.

Exclusion reason: No denominator or participation rate.

48. Korman N, Fox H, Skinner T, et al. Feasibility and Acceptability of a Student-Led Lifestyle (Diet and Exercise) Intervention Within a Residential Rehabilitation Setting for People with Severe Mental Illness, GO HEART (Group Occupation, Health, Exercise and Rehabilitation Treatment). *Frontiers in psychiatry Frontiers Research Foundation*. 2020;11:319. doi: 10.3389/fpsyt.2020.00319.

Exclusion reason: No denominator or participation rate.

49. Kwan RY, Lee D, Lee PH, Tse M, Cheung DS, Thiamwong L, Choi KS. Effects of an mHealth Brisk Walking Intervention on Increasing Physical Activity in Older People with Cognitive Frailty: Pilot Randomized Controlled Trial. *JMIR MHealth and UHealth*. 2020;8(7):e16596. doi: 10.2196/16596.

Exclusion reason: No denominator or participation rate.

50. Lando HA, Jeffery RW, McGovern PG, Forster JL, Baxter JE. Factors influencing participation in worksite smoking cessation and weight loss programs: The Healthy Worker Project. *Am J Health Promot*. 1993;8(1):22-4.

Exclusion reason: No denominator or participation rate.

51. Lee RE, McGinnis KA, Sallis JF, Castro CM, Chen AH, Hickmann SA. Active vs. passive methods of recruiting ethnic minority women to a health promotion program. *Ann Behav Med*. 1997;19(4):378-84.

Exclusion reason: No denominator or participation rate.

52. Lion A, Backes A, Duhem C, et al. Motivational Interviewing to Increase Physical Activity Behavior in Cancer Patients: A Pilot Randomized Controlled Trials. *Integr Cancer Ther*. 2020;19:1534735420914973. doi: 10.1177/1534735420914973.

Exclusion reason: No denominator or participation rate.

53. Lion A, Schummer C, Delagardelle C, Urhausen A, Seil R, Theisen D. Promotion of physical activity in patients with non-communicable diseases in Luxembourg: a follow-up of the Sport-Sante inventory from 2014. *Bull Soc Sci Med Grand Duche Luxemb*. 2016;(2):27-41.

Exclusion reason: No denominator or participation rate.

54. Lion A, Tchicaya A, Theisen D, Delagardelle C. Association between a national public health campaign for physical activity for patients with chronic diseases and the participation in Phase III cardiac rehabilitation in Luxembourg. *International Journal of Cardiology Heart & Vasculature*. 2021;32:100691. doi: 10.1016/j.ijcha.2020.100691.

Exclusion reason: No denominator or participation rate.

55. Lopez-Roman FJ, Tornel-Minarro FI, Delsors-Merida-Nicolich E, Fernandez-Lopez L, Martinez-Ros MT, Garcia Sanchez E, Lopez-Santiago A. Feasibility of implementing a preventive physical exercise programme recommended by general practitioners in cardiovascular risk patients: A pre-post comparison study. *European Journal of General Practice*. 2020;26(1):71-78. doi: 10.1080/13814788.2020.1760836.

Exclusion reason: No denominator or participation rate.

56. Markle-Reid M, Ploeg J, Fisher K, et al. The Aging, Community and Health Research Unit-Community Partnership Program for older adults with type 2 diabetes and multiple chronic conditions: a feasibility study. *Pilot & Feasibility Studies*. 2016;2:24.

Exclusion reason: No denominator or participation rate.

57. Marshall S, Taki S, Love P, et al. Feasibility of a culturally adapted early childhood obesity prevention program among migrant mothers in Australia: a mixed methods evaluation. *BMC Public Health*. 2021;21(1):1159. doi: 10.1186/s12889-021-11226-5.

Exclusion reason: No denominator or participation rate.

58. Matthews L, Mitchell F, Stalker K, et al. Process evaluation of the Walk Well study: a cluster-randomised controlled trial of a community based walking programme for adults with intellectual disabilities. *BMC Public Health*. 2016;16:527. doi: 10.1186/s12889-016-3179-6.

Exclusion reason: No denominator or participation rate.

59. McDonald PW. A practical, cost-effective method for recruiting people into healthy eating behavior programs. *Prev Chronic Dis*. 2007;4(2):A26.

Exclusion reason: No denominator or participation rate.

60. Mesquita CC, Ribeiro JC, Moreira P. An exercise program improves health-related quality of life of workers. *Applied Research in Quality of Life*. 2012;7(3):295-307. doi: 10.1007/s11482-011-9161-7.

Exclusion reason: Recruitment strategy not specified.

61. Metayer N, Boulos R, Tovar A, et al. Recruitment of New Immigrants into a Randomized Controlled Prevention Trial: The Live Well Experience. *J Prim Prev*. 2018;39(5):453-468. doi: 10.1007/s10935-018-0519-6.

Exclusion reason: No denominator or participation rate.

62. Michaud TL, Wilson K, Silva F, Almeida F, Katula J, Estabrooks P. Costing a population health management approach for participant recruitment to a diabetes prevention study. *Transl Behav Med*. 2021;11(10):1864-1874. doi: 10.1093/tbm/ibab054.

Exclusion reason: Recruitment strategy not specified.

63. Mikels JA, Young NA, Liu X, Stine-Morrow EAL. Getting to the Heart of the Matter in Later Life: The Central Role of Affect in Health Message Framing. *Gerontologist*. 2021;61(5):756-762. doi: 10.1093/geront/gnaa128.

Exclusion reason: No denominator or participation rate.

64. Napolitano MA, Harrington CB, Patchen L, et al. Feasibility of a Digital Intervention to Promote Healthy Weight Management among Postpartum African American/Black Women. *Int J Environ Res Public Health*. 2021;18(4):23. doi: 10.3390/ijerph18042178.

Exclusion reason: No denominator or participation rate.

65. Nhim K, Gruss SM, Porterfield DS, et al. Using a RE-AIM framework to identify promising practices in National Diabetes Prevention Program implementation. *Implementation Science*. 2019;14(1):81. doi: 10.1186/s13012-019-0928-9.

Exclusion reason: No denominator or participation rate.

66. O'Hara BJ, Bauman AE, King EL, Phongsavan P. Process evaluation of the advertising campaign for the NSW Get Healthy Information and Coaching Service. *Health Promot J Austr*. 2011;22(1):68-71.

Exclusion reason: No denominator or participation rate.

67. O'Hara BJ, Bauman AE, Phongsavan P. Using mass-media communications to increase population usage of Australia's Get Healthy Information and Coaching Service R. *BMC Public Health*. 2012;12:762. doi: 10.1186/1471-2458-12-762.

Exclusion reason: No denominator or participation rate.

68. O'Hara BJ, Eggins D, Phongsavan P, Milat AJ, Bauman AE, Wiggers J. Piloting proactive marketing to recruit disadvantaged adults to a community-wide obesity prevention program. *Public Health Research & Practice*. 2015;25(2):e2521521. doi: 10.17061/phrp2521521.

Exclusion reason: No denominator or participation rate.

69. O'Hara BJ, Phongsavan P, Rissel C, Hardy LL, Zander A, Greenaway M, Bauman AE. Role of general practice in the utilisation of the NSW Get Healthy Information and Coaching Service. *Australian Journal of Primary Health*. 2015;21(2):182-8.

Exclusion reason: No denominator or participation rate.

70. O'Hara BJ, Phongsavan P, Venugopal K, Bauman AE. Characteristics of participants in Australia's Get Healthy telephone-based lifestyle information and coaching service: reaching disadvantaged communities and those most at need. *Health Educ Res*. 2011;26(6):1097-106. doi: 10.1093/her/cyr091.

Exclusion reason: No denominator or participation rate.

71. Pettersson B, Bajraktari S, Skelton DA, Zingmark M, Rosendahl E, Lundin-Olsson L, Sandlund M. Recruitment strategies and reach of a digital fall-prevention intervention for community-dwelling older adults. *Digital Health*. 2022;8. doi: 10.1177/20552076221126050.

Exclusion reason: No denominator or participation rate.

72. Rasmussen M, Poulsen EK, Rytter AS, Kristiansen TM, Bak CK. Experiences with Recruitment of Marginalized Groups in a Danish Health Promotion Program: A Document Evaluation Study. *PLoS One*. 2016;11(6):e0158079. doi: 10.1371/journal.pone.0158079.

Exclusion reason: No denominator or participation rate.

73. Saez L, Langlois J, Legrand K, et al. Reach and Acceptability of a Mobile Reminder Strategy and Facebook Group Intervention for Weight Management in Less Advantaged Adolescents: Insights From the PRALIMAP-INES Trial. *JMIR MHealth and UHealth*. 2018;6(5):e110. doi: 10.2196/mhealth.7657.

Exclusion reason: No denominator or participation rate.

74. Salvy SJ, Carandang K, Vigen CL, et al. Effectiveness of social media (Facebook), targeted mailing, and in-person solicitation for the recruitment of young adult in a diabetes self-management clinical trial. *Clinical Trials*. 2020;17(6):664-674. doi: 10.1177/1740774520933362.

Exclusion reason: No denominator or participation rate.

75. Santiago-Torres M, Contento I, Koch P, et al. Mi Vida Saludable! A randomized, controlled, 2 x 2 factorial trial of a diet and physical activity intervention among Latina breast cancer survivors: Study design and methods. *Contemp Clin Trials*. 2021;110:106524. doi: 10.1016/j.cct.2021.106524.

Exclusion reason: No denominator or participation rate.

76. Shilts MK, Diaz Rios LK, Panarella KH, et al. Feasibility of Colocating a Nutrition Education Program into a Medical Clinic Setting to Facilitate Pediatric Obesity Prevention. *J Prim Care Community Health*. 2021;12:21501327211009695. doi: 10.1177/21501327211009695.

Exclusion reason: No denominator or participation rate.

77. Siddiqui M, Cooper LA, Appel LJ, et al. Recruitment and enrollment of African Americans and Caucasians in a health promotion trial for persons with serious mental illness. *Ethn Dis*. 2015;25(1):72-7.

Exclusion reason: No denominator or participation rate.

78. Simon D, Kaimal AJ, Oken E, Hivert MF. Reaching women with obesity to support weight loss before pregnancy: feasibility and qualitative assessment. *Therapeutic Advances in Reproductive Health*. 2020;14. doi: 10.1177/2633494120909106.

Exclusion reason: No denominator or participation rate.

79. Singh AS, Chinapaw MJ, Brug J, van Mechelen W. Process evaluation of a school-based weight gain prevention program: the Dutch Obesity Intervention in Teenagers (DOiT). *Health Educ Res*. 2009;24(5):772-7. doi: 10.1093/her/cyp011.

Exclusion reason: Recruitment strategy not specified.

80. Smit E, Leenaars K, Wagemakers A, van der Velden K, Molleman G. How to recruit inactive residents for lifestyle interventions: participants' characteristics based on various recruitment strategies. *Health Promot Int*. 2021;36(4):989-999. doi: 10.1093/heapro/daaa134.

Exclusion reason: No denominator or participation rate.

81. Smith AL. *Evaluation of a pilot workplace health promotion intervention targeting employees' health behaviours: The Motiv8 workplace series*. Queen's University (Canada); 2010.

Exclusion reason: Thesis/Dissertation.

82. Sofija E, Plugge M, Wiseman N, Harris N. 'This is the beginning of the new me': process evaluation of a group fitness intervention to promote wellbeing in formerly homeless individuals. *BMC Public Health*. 2018;18(1):290. doi: 10.1186/s12889-018-5175-5.

Exclusion reason: Not experimental or quasi-experimental.

83. Staffileno BA, Zschunke J, Weber M, Gross LE, Fogg L, Tangney CC. The Feasibility of Using Facebook, Craigslist, and Other Online Strategies to Recruit Young African American Women for a Web-Based Healthy Lifestyle Behavior Change Intervention. *J Cardiovasc Nurs*. 2017;32(4):365-371. doi: 10.1097/JCN.0000000000000360.

Exclusion reason: No denominator or participation rate.

84. Stockton MB, McClanahan BS, Lanctot JQ, Klesges RC, Beech BM. Identification of facilitators and barriers to participation in weight gain prevention research by African American girls. *Contemp Clin Trials*. 2012;33(1):38-45. doi: 10.1016/j.cct.2011.08.010.

Exclusion reason: No denominator or participation rate.

85. Story M, Sherwood NE, Obarzanek E, et al. Recruitment of African-American pre-adolescent girls into an obesity prevention trial: the GEMS pilot studies. *Ethn Dis*. 2003;13(1 Suppl 1):S78-87.

Exclusion reason: No denominator or participation rate.

86. Strunin L, Douyon M, Chavez M, Bunte D, Horsburgh CR. The GirlStars program: challenges to recruitment and retention in a physical activity and health education program for adolescent girls living in public housing. *Prev Chronic Dis*. 2010;7(2):A42.

Exclusion reason: No denominator or participation rate.

87. Swaine J, Parish SL, Luken K, Atkins L. Recruitment and consent of women with intellectual disabilities in a randomised control trial of a health promotion intervention. *J Intellect Disabil Res*. 2011;55(5):474-83. doi: 10.1111/j.1365-2788.2011.01399.x.

Exclusion reason: No denominator or participation rate.

88. Taverno Ross SE, Smith Tapia I, Saunders RP, Documet PI, Pate RR. Implementation Monitoring of a Promotora-Led, Home-Based Obesity Prevention Pilot Study with Latino Preschool Children and Their Mothers. *Int Q Community Health Educ*. 2021;41(4):411-418. doi: 10.1177/0272684X20970375.

Exclusion reason: Recruitment strategy not specified.

89. Taylor-Piliae RE, Froelicher ES. Methods to optimize recruitment and retention to an exercise study in Chinese immigrants. *Nurs Res*. 2007;56(2):132-6.

Exclusion reason: No denominator or participation rate.

90. Thogersen-Ntoumani C, Quested E, Biddle SJH, et al. Trial feasibility and process evaluation of a motivationally-embellished group peer led walking intervention in retirement villages using the RE-AIM framework: the residents in action trial (RiAT). *Health Psychology & Behavioral Medicine*. 2019;7(1):202-233. doi: 10.1080/21642850.2019.1629934.

Exclusion reason: No denominator or participation rate.

91. Toft UN, Kristoffersen LH, Aadahl M, von Huth Smith L, Pisinger C, Jorgensen T. Diet and exercise intervention in a general population--mediators of participation and adherence: the Inter99 study. *Eur J Public Health*. 2007;17(5):455-63.

Exclusion reason: No denominator or participation rate.

92. Tripicchio GL, Grady Smith J, Armstrong-Brown J, et al. Recruiting Community Partners for Veggie Van: Strategies and Lessons Learned from a Mobile Market Intervention in North Carolina, 2012-2015. *Prev Chronic Dis*. 2017;14:E36. doi: 10.5888/pcd14.160475.

Exclusion reason: No denominator or participation rate.

93. Tully L, Spyreli E, Allen-Walker V, et al. Recruiting 'hard to reach' parents for health promotion research: experiences from a qualitative study. *BMC Res Notes*. 2021;14(1):276. doi: 10.1186/s13104-021-05653-1.

Exclusion reason: No denominator or participation rate.

94. van Nassau F, Singh AS, Hoekstra T, van Mechelen W, Brug J, Chinapaw MJ. Implemented or not implemented? Process evaluation of the school-based obesity prevention program DOiT and associations with program effectiveness. *Health Educ Res*. 2016;31(2):220-33. doi: 10.1093/her/cyw007.

Exclusion reason: No denominator or participation rate.

95. Vangeepuram N, Townsend K, Arniella G, Goytia C, Horowitz CR. Recruitment in clinical versus community-based sites for a pilot youth diabetes prevention program, East Harlem, New York, 2011–2012. *Preventing Chronic Disease: Public Health Research, Practice, and Policy*. 2016;13.

Exclusion reason: No denominator or participation rate.

96. Velott DL, Baker SA, Hillemeier MM, Weisman CS. Participant recruitment to a randomized trial of a community-based behavioral intervention for pre- and interconceptional women findings from the Central Pennsylvania Women's Health Study. *Womens Health Issues*. 2008;18(3):217-24. doi: 10.1016/j.whi.2008.02.002.

Exclusion reason: No denominator or participation rate.

97. Vetrovsky T, Cupka J, Dudek M, Kuthanova B, Vetrovska K, Capek V, Bunc V. A pedometer-based walking intervention with and without email counseling in general practice: a pilot randomized controlled trial. *BMC Public Health*. 2018;18(1):635. doi: 10.1186/s12889-018-5520-8.

Exclusion reason: No denominator or participation rate.

98. Villalobos A, Alaimo K, Erickson C, et al. CAPS on the move: Crafting an approach to recruitment for a randomized controlled trial of community gardening. *Contemporary Clinical Trials Communications*. 2019;16:100482. doi: 10.1016/j.conctc.2019.100482.

Exclusion reason: No denominator or participation rate.

99. Vincent D, Pasvogel A, Barrera L. A feasibility study of a culturally tailored diabetes intervention for Mexican Americans. *Biol Res Nurs*. 2007;9(2):130-41.

Exclusion reason: No denominator or participation rate.

100. Vollmer WM, Svetkey LP, Appel LJ, et al. Recruitment and retention of minority participants in the DASH controlled feeding trial. DASH Collaborative Research Group. Dietary Approaches to Stop Hypertension. *Ethn Dis*. 1998;8(2):198-208.

Exclusion reason: No denominator or participation rate.

101. Wallace JI, Buchner DM, Grothaus L, Leveille S, Tyll L, LaCroix AZ, Wagner EH. Implementation and effectiveness of a community-based health promotion program for older adults. *Journals of Gerontology Series A-Biological Sciences & Medical Sciences*. 1998;53(4):M301-6.

Exclusion reason: No denominator or participation rate.

102. Webb F, Doldren M, Blanchard S, Brennan VM, Kumanyika SK, Zambrana RE. *Winning Over Weight Wellness: A culturally relevant, interactive health program for underserved faith-based groups*. Obesity interventions in underserved communities: Evidence and directions. Johns Hopkins University Press; 2014:214-220.

Exclusion reason: No denominator or participation rate.

103. Wilbur J, McDevitt J, Wang E, et al. Recruitment of African American women to a walking program: eligibility, ineligibility, and attrition during screening. *Res Nurs Health*. 2006;29(3):176-89.

Exclusion reason: No denominator or participation rate.

104. Williams A, Mason A, Wold J. Cultural sensitivity and day care workers: examination of a worksite based cardiovascular disease prevention project. *AAOHN J*. 2001;49(1):35-43.

Exclusion reason: No denominator or participation rate.

105. Wilson KE, Estabrooks PA. Does environmental message framing impact proportional reach and sample representativeness related to motivational characteristics? *Transl Behav Med*. 2022;12(4):585-594. doi: 10.1093/tbm/ibab124.

Exclusion reason: Intervention not in content area.

106. Wisk LE, Nelson EB, Magane KM, Weitzman ER. Clinical Trial Recruitment and Retention of College Students with Type 1 Diabetes via Social Media: An Implementation Case Study. *J Diabetes Sci Technol*. 2019;13(3):445-456. doi: 10.1177/1932296819839503.

Exclusion reason: No denominator or participation rate.

107. Yoong SL, Wolfenden L, Finch M, Williams A, Dodds P, Gillham K, Wyse R. A randomised controlled trial of an active telephone-based recruitment strategy to increase childcare-service staff attendance at a physical activity and nutrition training workshop. *Health Promot J Austr*. 2013;24(3):224-6. doi: 10.1071/HE13055.

Exclusion reason: Intervention not in content area.

108. Zigmont VA, Shoben AB, Kaye GL, Snow RJ, Clinton SK, Harris RE, Olivo-Marston SE. An Evaluation of Reach for a Work Site Implementation of the National Diabetes Prevention Program Focusing on Diet and Exercise. *Am J Health Promot*. 2018;32(6):1417-1424. doi: 10.1177/0890117117733348.

Exclusion reason: No denominator or participation rate.
