# Supplementary material for: Identifying recruitment strategies to improve the reach of evidence-based health promotion, disease prevention, and disease self-management interventions: a scoping review
Source: Front Public Health. 2025 Apr 1;13:1515042. doi: 10.3389/fpubh.2025.1515042 (PMC12023269; doi:10.3389/fpubh.2025.1515042)
Supplement: Supplementary file 5 [file Supplementary_file_5.docx]

Appendix E: Differences in methods between the protocol and manuscript

Documentation history:

10/2024

While reviewing titles and abstracts, we encountered many articles that were testing strategies to improve the reach and enrollment to a clinical trial. We added this as exclusion criteria because the focus of this review was recruitment strategies to health promotion programs.

We also did not report Study Type as it related to the lifestyle intervention as it added confusion for authors when considering the study design (e.g., RCT vs Observational) related to how the recruitment strategies were tested.

09/2023

Under “Other information”, the data element study design was consistently confusing for reviewers. We consolidated the options to “RCT or observational”

07/2023

While attempting to abstract the PRISM/RE-AIM factors, we found that there were few articles that reported on these elements. We revised our abstraction to report “Did they use PRISM? [yes/no]”, “Did they use RE-AIM? [yes/no]”, “Did they report on the degree of recruitment strategy completion as intended? [yes/no]” If an element was reported, we abstracted the relevant information.
